# Deuterated Water Accelerates Phase-Separated Droplet Formation and Enables Directional Motion

**DOI:** 10.1021/jacs.6c03395

**Published:** 2026-06-09

**Authors:** Caihong Lin, Jingjing Yu, Dawei Qi, Xuncheng Shi, Tuomas Niemi-Aro, Jianwei Li

**Affiliations:** † MediCity Research Laboratory, 8058 University of Turku, Tykistökatu 6, 20520 Turku, Finland; ‡ NMR Research Unit, Faculty of Science, 6370 University of Oulu, Pentti Kaiteran katu 1, 90014 Oulu, Finland; § Institute of Biotechnology, Helsinki Institute of Life Science, University of Helsinki, Viikinkaari 1, 00014 Helsinki, Finland; ∥ Macao Institute of Materials Science and Engineering (MIMSE), Faculty of Innovation Engineering, 58816 Macau University of Science and Technology, Taipa, 999078 Macao, China

## Abstract

The spatiotemporal coordination of compartment formation and directed transport is fundamental to the cellular organization. However, replicating these coupled behaviors in fully aqueous synthetic systems remains challenging. We report an isotopic solvent signaling strategy that leverages the physicochemical differences between deuterated water (D_2_O) and light water (H_2_O) to control the liquid–liquid phase separation (LLPS) and motility of dynamic covalent droplets. Our system utilizes the *in situ* generation of cationic imine surfactants that complex with anionic macrocycles to form coacervate droplets. We demonstrate that D_2_O significantly accelerates droplet formation compared to that of H_2_O by promoting early association and amplifying the hydrophobic interactions associated with imine surfactants. Furthermore, by establishing a spatial H_2_O/D_2_O gradient, we trigger a surface-tension imbalance that drives the directional transport of droplets from D_2_O-rich to H_2_O-rich regions via Marangoni flow. These motile droplets can move faster than that without an isotope gradient and perform complex functions using fluorescent dyes as a demonstration. These functions include autonomous cargo transport and chemical exchange with their surroundings during migration. This work establishes isotopic substitution as a powerful and noninvasive trigger for governing supramolecular assembly and motility. It offers a new dimension for engineering adaptive, lifelike soft matter.

## Graphical abstract

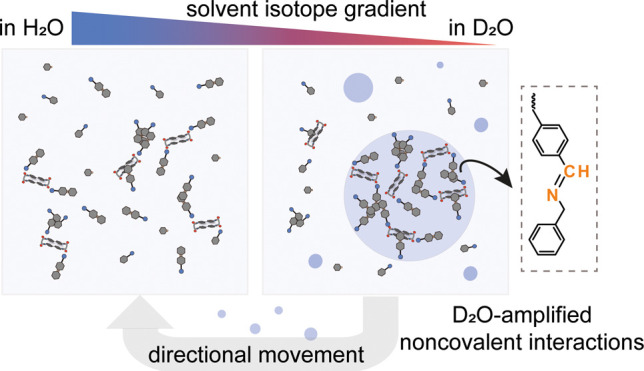

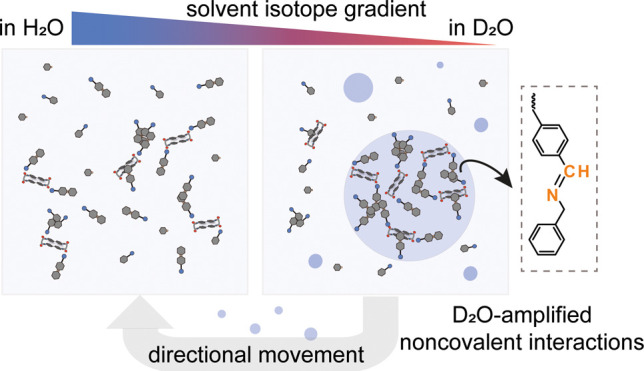

## 1 Introduction

Living cells organize chemistry in space and time by forming dynamic, membrane-free compartments in a crowded aqueous milieu^1–4^ and redistributing them through directed transport.^5–9^ This coordination of localized compartment formation and biased transport is central to regulation in cellular physiological activities.^10–14^ For example, DNA-rich condensates move directionally toward the mitotic spindle during cell division,^14^ and synaptic vesicles display short-distance directed motion triggered by Ca^2+^ sensing.^8^ Reproducing this coupled behavior in fully aqueous synthetic systems opens routes to life-like functions such as adaptive substance handling and compartmental trafficking.^15–18^

Liquid–liquid phase separation (LLPS) offers an attractive route to aqueous compartments because it spontaneously forms liquid condensed phase from aqueous solution through multiple noncovalent interactions.^19^ In associative LLPS, oppositely charged molecules complex to yield compartment-like droplets.^20–22^ A major challenge, however, is that triggers commonly used to induce LLPS are not easily repurposed to simultaneously guide droplet motion in fully aqueous environments, because this requires establishing a responsive connection between LLPS components and their solvent environment. Directed droplet motion is often achieved by establishing chemical gradients that drive transport, but practical gradients are largely limited to ion, pH, or surfactant concentration,^23–27^ and most demonstrations rely on immiscible oil/water droplets^23,28–30^ rather than all-aqueous phase-separated compartments. These constraints motivate an alternative strategy in which the solvent itself provides the stimulus for both droplet formation and transport.

To this end, we introduce isotopic-solvent control using heavy water (D_2_O) and light water (H_2_O), which differ subtly yet meaningfully in physicochemical properties.^31–33^ On one hand, D_2_O forms a stronger hydrogen bonding network than H_2_O,^34,35^ which could amplify noncovalent interactions in water-rich media and thereby lower the barrier to LLPS. Consistent with this view, replacing solvent H_2_O with D_2_O has been found to enhance hydrophobic effect across supramolecular assemblies of biomacromolecules, polymers, and small-molecular surfactants, and to accelerate processes that proceed via hydrophobic kinetic intermediates.^36–38^ On the other hand, a built-in composition gradient is provided when D_2_O and H_2_O coexist. Because D_2_O has a lower surface tension than H_2_O,^39^ such gradient could generate interfacial tension imbalance and drive Marangoni flow from D_2_O-rich to H_2_O-rich regions. Taken together, these features suggest that water isotope pair could offer the opportunity to integrate selective droplet formation with inherent directional motion without changing ionic strength, pH, or adding external fuels. Previous reports on isotope-governed phase separation remained limited to proteins and polyelectrolytes,^40,41^ probably because strong and rapid complexation between solutes could impair sensitivity to isotopic solvent. To access D_2_O sensitivity in synthetic LLPS system, we embed dynamic imine chemistry as a programmable motif. Reversible imine formation (–C=N–) eliminates only water (H_2_O) as a byproduct^42^ and could tune hydrophilic–hydrophobic balance of building blocks while promoting assembly through hydrophobic aggregation.^43–46^ Given the reported enhancement of hydrophobic effect in D_2_O, imine linkages could be a promising motif to encode solvent-isotope sensitivity into LLPS.

In this work, we demonstrate that D_2_O promotes the formation of an associative LLPS droplet and enables their directional motion toward H_2_O-rich regions. We designed an imine-mediated synthetic system in which a cationic imine surfactant is generated reversibly in situ (Scheme 1). Raising the D_2_O fraction favors early association and strengthens hydrophobic interactions among imine surfactants to stabilize the condensed phase. In contrast, weak hydrophobic enhancement in H_2_O leaves more early formed species solubilized, suppressing LLPS. A spatial H_2_O–D_2_O gradient then drives directional flow of the droplets toward H_2_O-rich, higher surface tension regions, enabling cargo transport (Nile red) and substance exchange (methylene blue) during motion. Overall, this study adopts isotopic solvent control to couple droplet growth and guided motility in an aqueous-phase-separated system, providing new insights for engineering more advanced and complex lifelike synthetic systems.

**Scheme 1:**
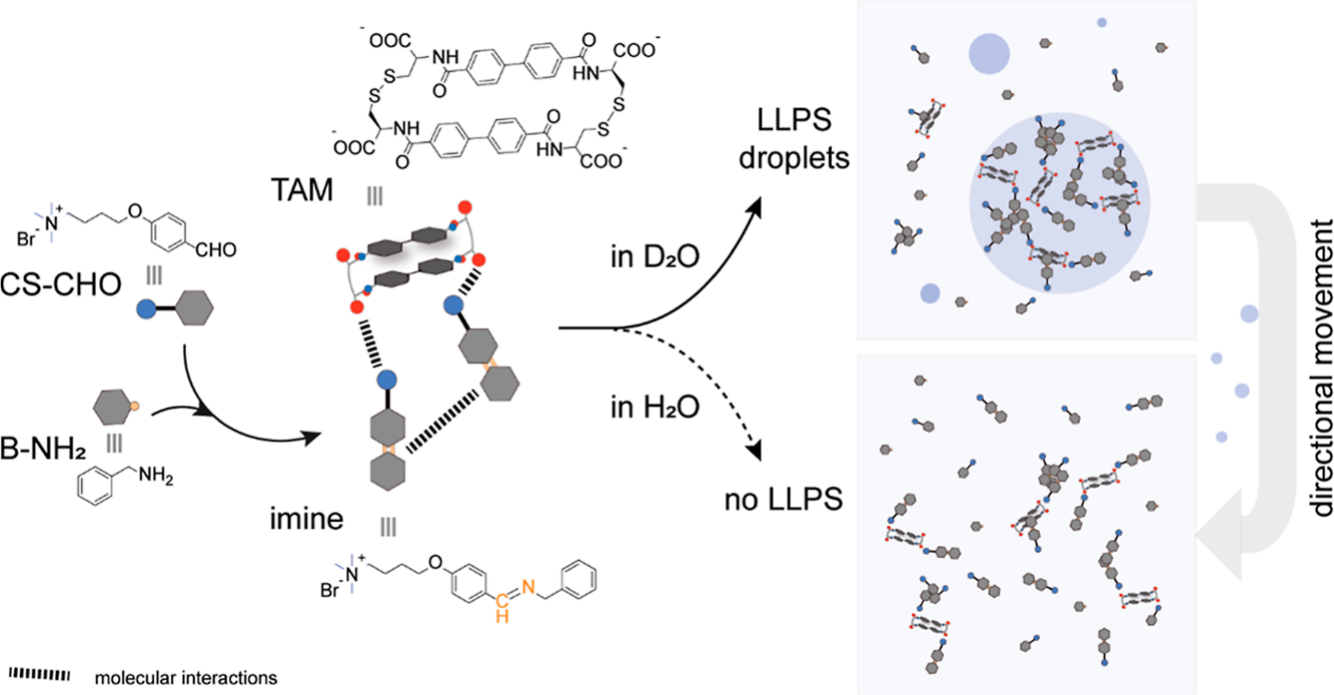
Design of an Interactive Multicomponent System Composed of Tetracarboxylic Acid Macrocycle (TAM), Aldehyde-modified Cationic Surfactant (CS-CHO), Water-Soluble Benzylamine (B-NH_2_), and In Situ Produced Imine Surfactant via Dynamic Covalent Reaction, and Cartoon Illustrations Showing D_2_O-Promoted Phase Separation Relative to H_2_O Buffer and Directional Movement of Droplets From D_2_O-Rich to H_2_O-Rich Regions (pH or pD = 11.4)

## 2 Results and Discussion

### 2.1 Design of Phase-Separated and Motile Droplets Govern by D_2_O

To enable D_2_O-governed droplet growth and directional motion, the designed phase-separated synthetic system is modified from our previous small-molecule LLPS study^47^ and composed of tetracarboxylic acid macrocycle (**TAM**), aldehyde-modified cationic surfactant (**CS-CHO**), and benzylamine (**B-NH**_2_) (Scheme 1). TAM is chosen in this study because its tetracarboxylate structure provides multiple electrostatic binding sites for the cationic surfactant, while its biphenyl linkers also contribute additional aromatic interactions. Both characteristics of TAM facilitate phase separation when it forms complexes with cationic surfactant. To maintain sufficient nucleophilicity of B-NH_2_ for imine formation, all samples were prepared in 120 mM phosphate buffer with pH or pD equal to 11.4, unless otherwise specified. For deuterated buffers, the pD values were corrected according to pD = pH_read_ + 0.4 for reducing the difference in acidity compared with H_2_O. In situ, CS-CHO and B-NH_2_ react reversibly to yield a cationic imine surfactant with enhanced hydrophobicity. LLPS behavior and reaction efficiency were first tested in a mixture of CS-CHO and B-NH_2_ with equal initial concentrations of 20 mM (Figure S9). No LLPS was detected in this mixture, as indicated by constant absorbance during the turbidity-tracking process. Importantly, the apparent imine yield of this mixture rapidly reached a maximum of 24.4% in the first 40 min and then remained stable. Such massive and quick production of cationic imine surfactant provides an opportunity for complexation with anionic TAM to trigger LLPS. Because D_2_O is able to amplify noncovalent interactions, increasing the D_2_O fraction is expected to lower the LLPS barrier and promote droplet formation by strengthening interactions among TAM and imine assemblies. Moreover, when D_2_O and H_2_O coexist, a natural gradient between D_2_O and H_2_O is ready to drive passive diffusion of these droplets. Therefore, this study aims to use isotopic solvent control for coupling localized formation with directed transport within a synthetic LLPS system.

### 2.2 D_2_O-Promoted Droplet Formation

To verify the possibility of D_2_O to promote LLPS droplet formation in the proposed synthetic system, we prepared a series of samples by mixing x mM TAM, y mM CS-CHO, and z mM B-NH_2_ in either D_2_O or H_2_O phosphate buffer, denoted as **D/H x-y-z**, respectively. Turbidity tracking was performed on all prepared samples, and the collected data were plotted into partial phase diagrams (Figures 1a and S10). Typically, an increased turbidity means the emergence of LLPS droplets, as they scatter more light. As shown in Figure 1a, LLPS occurs only within a defined composition region. In H_2_O, droplets were observed only at 20 mM CS-CHO/B-NH_2_ with 2.5 or 5.0 mM TAM, whereas in D_2_O, the droplet region extended to 15 mM CS-CHO/B-NH_2_ at the same TAM/CS-CHO/B-NH_2_ ratios. This composition dependence suggests that LLPS arises from a balance of multicomponent associative interactions. In addition, D_2_O shifts the phase boundary toward lower precursor concentrations, thereby broadening the composition window for the droplet formation.

**Figure 1:**
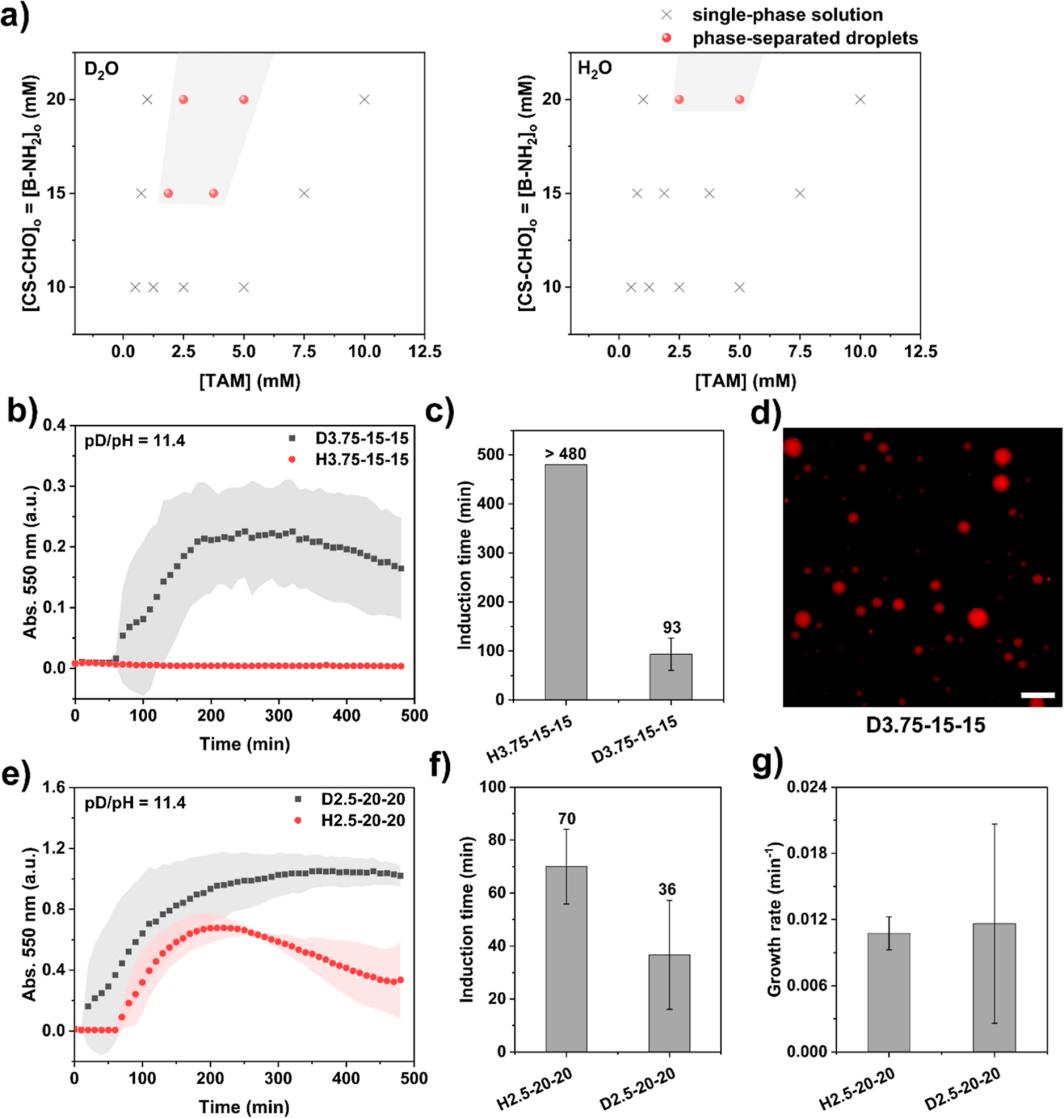
Phase-separated droplet formation promoted by deuterated water. (a) Partial phase diagrams of the TAM/CS-CHO-B-NH_2_ system in D_2_O buffer (left) and H_2_O buffer (right) at varying molar ratios and concentrations. Gray regions indicate compositions that formed phase-separated droplets within 8 h, and red dots mark the specific compositions examined in detail. Black crosses denote single-phase solutions. (b) Time-dependent turbidity of H3.75-15-15 and D3.75-15-15 and (c) comparison of their induction time. Induction time is defined as the elapsed time before the turbidity starts to increase. Data are shown as the mean ± standard deviation (*n* = 3). The shaded region indicates the standard deviation. (d) Microscopic morphology of sample D3.75-15-15 stained with Nile Red. Scale bar is 20 μm. (e) Time-dependent turbidity of H2.5-20-20 and D2.5-20-20 and comparisons of their (f) induction time and (g) growth rate. Growth rate is calculated from change in turbidity over the following 30 min. Data are shown as the mean ± standard deviation (*n* = 3). The shaded region indicates the standard deviation.

Turbidity tracking clearly demonstrated the kinetic difference between H_2_O and D_2_O buffers, as seen for both 3.75-15-15 and 2.5-20-20 (Figure 1b,e). A specific quantitative comparison was completed by analyzing their induction time and growth rate extracted from turbidity profiles in H_2_O and D_2_O buffers. D3.75-15-15 showed an induction time of 93 min and a measurable growth rate of 0.0041 min^–1^, whereas no obvious turbidity growth was detected for the corresponding H_2_O sample within the measurement time (Figures 1c and S11). For samples 2.5-20-20, induction time decreased from 70 min in H_2_O to 36 min in D_2_O, while the growth rates were similar (Figure 1f,g). These results show that the most significant kinetic difference between H_2_O and D_2_O buffers is the markedly shortened induction time in D_2_O, supporting the accelerated phase separation in the deuterated medium.

To confirm LLPS droplet formation, Nile Red was added as dye for visualizing under confocal microscope. After staining with Nile Red, well-defined, micron-sized spherical droplets were visualized in both D3.75-15-15 and D2.5-20-20, whereas small irregular dye aggregates formed in H3.75-15-15 (Figures 1d and S12). Dynamic light scattering (DLS) analysis also showed larger average hydrodynamic diameter of 1.5 μm in D3.75-15-15 than in H3.75-15-15. The occurrence of LLPS droplet formation made D3.75-15-15 have a higher absolute value in Zeta potential than H3.75-15-15 (Figure S11).

### 2.3 Droplet Formation Mediated by Imine Chemistry

To receive a detailed understanding of droplet formation, we identified the component participating in LLPS through Nuclear magnetic resonance (NMR). As LLPS-driven molecular assembly would change electron cloud density around protons, this will manifest in different time-dependent chemical shift changes. Accordingly, we tracked the chemical shift change of representative D3.75-15-15 right after preparation. It turned out that the chemical shift of imine proton (–CH=N–) showed a tendency to shift toward upfield (Δ > 0.15 ppm) along with the emergence of LLPS, while proton of aldehyde group (–CHO) exhibited unchanged chemical shift with time (Figure 2a). Analogous chemical shift changes of imine proton and aldehyde group were also observed for D2.5-20-20 (Figure S13). This result suggested that the interaction changes associated with LLPS are more pronounced for the imine surfactant than for the precursor CS-CHO. As spectral signals overlap during phase separation, complicating direct analysis of TAM and B-NH_2_, we complemented Nuclear Overhauser Effect spectroscopy (NOESY) measurement to explore the assembled mode in LLPS. From NOESY 2D plot, a strong correlation between imine surfactant and TAM was observed, whereas no such correlation with TAM was found for either CS-CHO or B-NH_2_ (Figure S14). When the LLPS droplets were isolated by centrifugation, we compared the signal integrals of supernatant and original sample. The results showed that the integrals associated with imine surfactant and TAM decreased by nearly half and 23%, respectively, while the integrals associated with CS-CHO merely decreased by 5% (Figure S15), indicating preferential enrichment of imine surfactant and TAM in the droplet phase. It is further validated that LLPS is driven by the complexation between imine surfactant and TAM.

**Figure 2:**
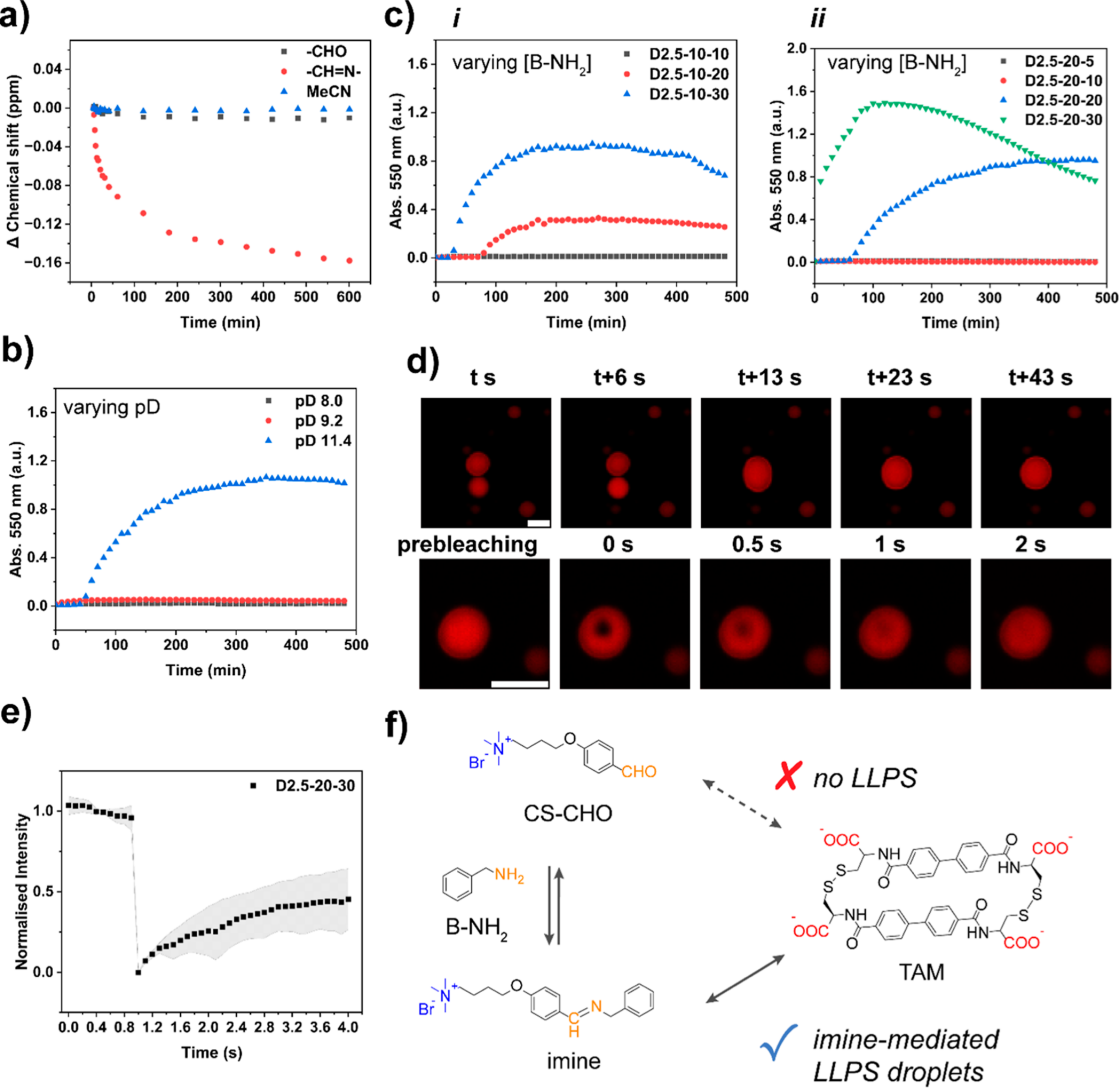
Droplet formation mediated by imine chemistry. (a) Time-dependent chemical shift change of the –CHO and –CH=N– peaks relative to their initial positions in proton spectra of D3.75-15-15 (500 MHz). The added MeCN was used as internal reference for chemical shift change. (b) pD-dependent turbidity test for D2.5-20-20. (c) B-NH_2_-dependent turbidity test for this multicomponent system at fixed TAM/CS-CHO concentration ratios of (i) 2.5 mM: 10 mM and (ii) 2.5 mM: 20 mM in D_2_O buffer. Results showed that increasing B-NH_2_ concentration promoted imine formation via reversible reaction, leading to droplet formation. (d) Time-lapse images during droplet fusion (top) and FRAP recovery (bottom) for D2.5-20-30. Scale bars are 10 μm. (e) Normalized FRAP recovery curve of D2.5-20-30 from three independent experiments. The shaded region indicates the standard deviation. (f) Schematic illustration of interactions among components in this multicomponent system, highlighting phase separation driven by interactions between produced cationic imine surfactant and anionic TAM.

Although CS-CHO and the imine surfactant share the same cationic backbone, LLPS droplets could not form in the binary system of TAM and CS-CHO, as seen from turbidity results (Figure S16). Compared with CS-CHO, the imine surfactant contains an additional benzyl-derived aromatic fragment, making it less hydrated and shifting the balance toward hydrophobic association. Therefore, the imine surfactant exhibits sufficient hydrophobicity bias to cross the LLPS threshold with TAM, whereas TAM/CS-CHO remains in the single-phase region.

To clearly illustrate the role of imine chemistry in LLPS droplet formation, we next perturbed the extent of imine production by varying solution pD or concentration of B-NH_2_. D2.5-20-20 was selected for the test because of its strongest turbidity response in the partial phase diagram at pD 11.4. Then, samples with the same composition concentration were prepared at different pD values (8.0, 9.2, and 11.4), and their imine yields and turbidity changes were collected. As expected, with lowered solution pD, B-NH_2_ was trapped by protonation and became less nucleophilic, leading to the observed significant decline in imine yield (Figure S17). As TAM remained essentially in the same deprotonation state within the investigated pD range, supported by minimal chemical-shift changes and titration result (Figure S18), a diminished kinetic turbidity at lower pD suggested the pD-dependent LLPS behavior was likely correlated with the extent of imine formation (Figure 2b). In addition, B-NH_2_-dependent turbidity tests were conducted at fixed pD 11.4, while keeping the other components constant. As seen from the turbidity curves, increasing the concentration of B-NH_2_ shortened the induction time and increased the turbidity amplitude, indicating that elevating imine formation accelerated nucleation and enhanced the extent of phase separation (Figure 2c). The decrease in turbidity observed for D2.5-20-30 was mainly attributed to droplet coalescence (Figure 2d) and sedimentation. These sedimented droplets had their internal fluidity, as evident by fast fluorescence recovery after photobleaching (FRAP) (Figure 2d,e).

Collectively, these results confirm that LLPS droplet formation is mediated by an in situ generated cationic imine surfactant complexing with negatively charged TAM and that the extent of imine formation provides a direct handle over droplet nucleation and growth (Figure 2f). The generated imine surfactant therefore provides a plausible molecular basis for the different phase separation kinetics observed in D_2_O and H_2_O.

### 2.4 D_2_O-Enhanced Noncovalent Interactions

Having identified the essential components responsible for LLPS, we first explored whether the isotope effect originated from differences in imine equilibrium at working pH/pD. Before analysis by liquid chromatography, samples were quenched by excess sodium borohydride (NaBH_4_), which reduced aldehyde and imine to their corresponding nondynamic products.^41^ It was found that H3.75-15-15 (no LLPS) and D3.75-15-15 (LLPS) showed identical imine yield kinetics, with peak values attained within the initial 30 min followed by a stable plateau (Figure 3a). This result indicated that D_2_O as a solvent did not largely affect the imine production in quantity and kinetics.

**Figure 3:**
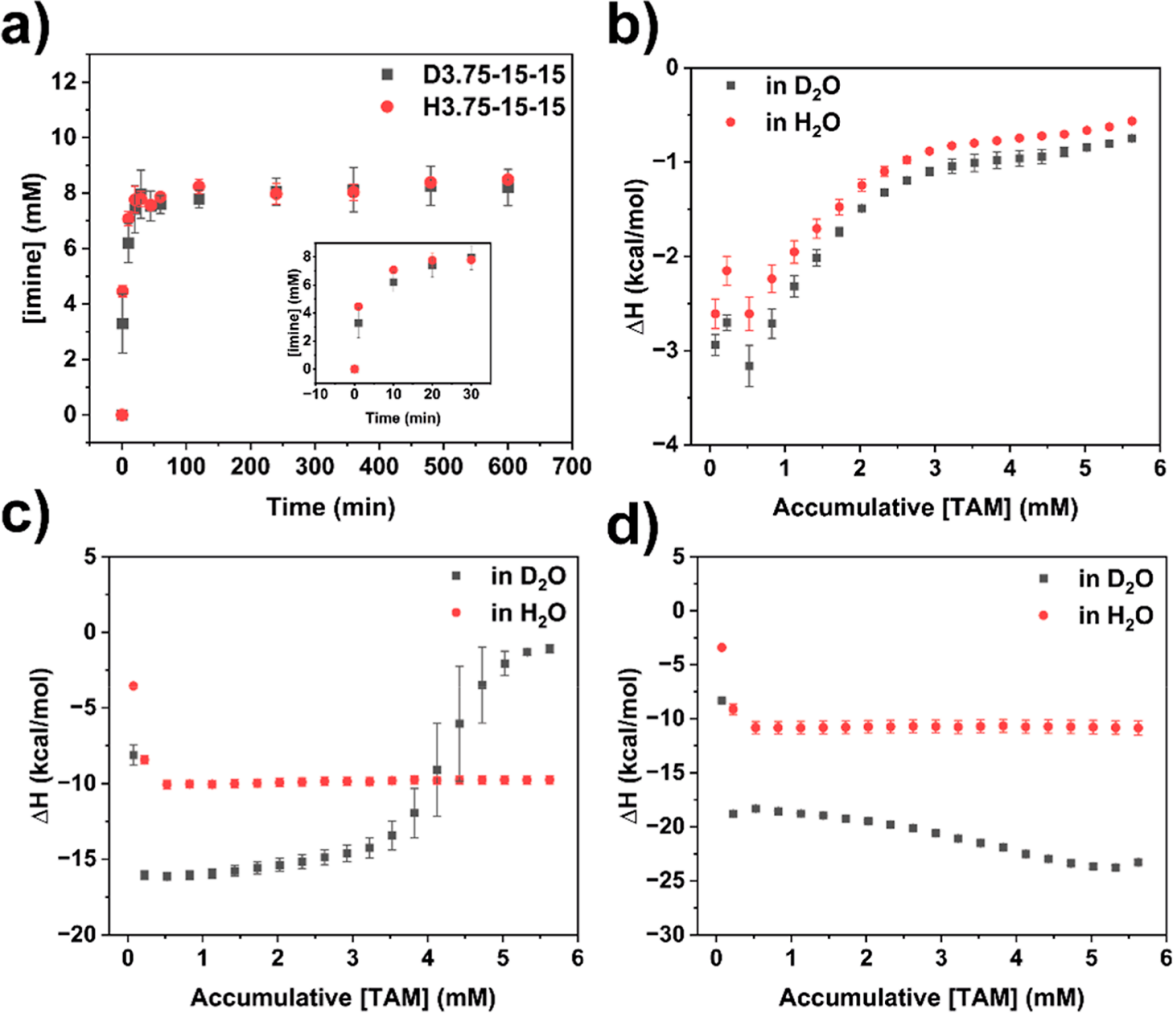
(a) Kinetic profiles of imine yields for D3.75-15-15 and H3.75-15-15, determined by ultra performance liquid chromatography (UPLC) after NaBH_4_ reduction. The enlarged inset shows the imine yield for the first 30 min. Data are shown as the mean ± standard deviation (*n* = 3). Enthalpy change from titration of TAM into (b) equilibrated B-NH_2_/CS-CHO solution (15 mM: 15 mM), (c) 6.5 mM CS-CHO solution, and (d) 6.5 mM B-NH_2_ solution in D_2_O and H_2_O buffer, respectively. The dilution heat of TAM was subtracted. Data are shown as the mean ± standard deviation (*n* = 3).

As LLPS was confirmed to be triggered by the complexation between TAM and imine surfactant, their interactions were further compared in D_2_O and H_2_O using isothermal calorimetric titration (ITC). Associative LLPS driven by oppositely charged molecules theoretically follows a nucleation–growth mechanism in which an initial nucleated phase forms once the energy barrier is overcome.^19^ Because the ITC measurements were performed under vigorous stirring and within a relatively short time scale, they were most informative about early association or nucleation. As depicted in Figure 3b, successive injections of TAM into an imine-containing equilibrium solution generated exothermic signals, with a larger enthalpy change observed in D_2_O buffer than in H_2_O buffer. We interpret this result as evidence for more favorable early association, specifically electrostatic complexation, that forms more stable phase-separated nuclei in the deuterated medium.

To reveal the possible contribution of unreacted components to the observed enthalpy change, control titrations of TAM into CS-CHO and B-NH_2_ solutions corresponding to the estimated residual concentrations were also completed. Both controls produced measurable exothermic signals and showed larger overall heat release in D_2_O than in H_2_O. For 6.5 mM CS-CHO, the titration profile in a D_2_O buffer showed a sigmoidal curve, suggesting a specific association process, whereas the nearly constant exothermic signal in a H_2_O buffer indicated a continuous and unsaturated heat-releasing process (Figure 3c). For 6.5 mM B-NH_2_, an unsaturated exothermic process was observed in both buffers (Figure 3d). However, the titration profile of the equilibrated imine-containing mixture differed from those of control systems based on the exothermic signals and inflection points. Smaller overall exothermic signals suggested that early association may be accompanied by the endothermic process, such as aggregate dissociation. Additionally, an earlier inflection point was likely attributed to favorable association with the imine surfactant. This favorable complexation of TAM and imine surfactant in D_2_O buffer was also supported by their closer diffusion coefficients relative to those in H_2_O buffer (Figure S19).

In addition to the contribution of favorable electrostatic complexation to D_2_O-accelerated LLPS, we reasoned that stronger hydrogen-bonding network of D_2_O may result in the weakened solvent’s affinity to solutes and this reduction of solvation dynamics could further drive enhanced intermolecular association. To verify this, we next examined how D_2_O altered the local molecular environment during phase separation. A series of samples H-D 3.75-15-15 with varying D_2_O content (0%, 25%, 50%, 75%, and 100%) was prepared and allowed for equilibrium. These equilibrated samples showed a monotonic increase in turbidity with D_2_O fraction (Figure S20).

Deuterium-dependent proton chemical shift changes were analyzed and displayed in Figures 4a and S21. When D_2_O content was higher, the chemical shift of imine proton (–CH=N–, g′) underwent a significant shift toward upfield, indicating enhanced electronic shielding environment. In addition, the benzylic proton (i′), protons on the aliphatic part (d′, b′), and proton on the hydrophilic part (a′) exhibited weakened chemical shift change in turn. In contrast, protons of TAM did not display the same significant changes in chemical shift as imine surfactant (Figure S22). Along with the increased D_2_O content, chemical shifts of aromatic protons (1′ and 2′) remained basically unchanged, and the aliphatic proton (4′) close to carboxyl group presented a slight chemical shift change toward upfield, with an amplitude less than half that observed for imine proton (g′). It could be inferred that local microenvironment surrounding hydrophobic aromatic domain of imine surfactant was preferentially altered, hinting hydrophobic aggregation promoted by D_2_O. When interpreting the result of chemical shift change, control measurements on TAM alone and on binary B-NH_2_/CS-CHO system at different D_2_O fractions were used to evaluate the possibility of aggregation effect by themselves. Results showed only negligible D_2_O-dependent chemical-shift changes (<0.02 ppm for TAM and <0.01 ppm for selected imine-surfactant protons in the binary system) (Figures S23 and S24a). Therefore, much larger chemical shift changes observed in the TAM/B-NH_2_/CS-CHO system are associated with molecular crowding and assembly during LLPS. Imine proton could be regarded as a sensitive reporter of D_2_O-dependent microenvironmental changes because of its pronounce chemical shift change. This change was attributed to the synergy of electrostatic and hydrophobic interactions.

**Figure 4:**
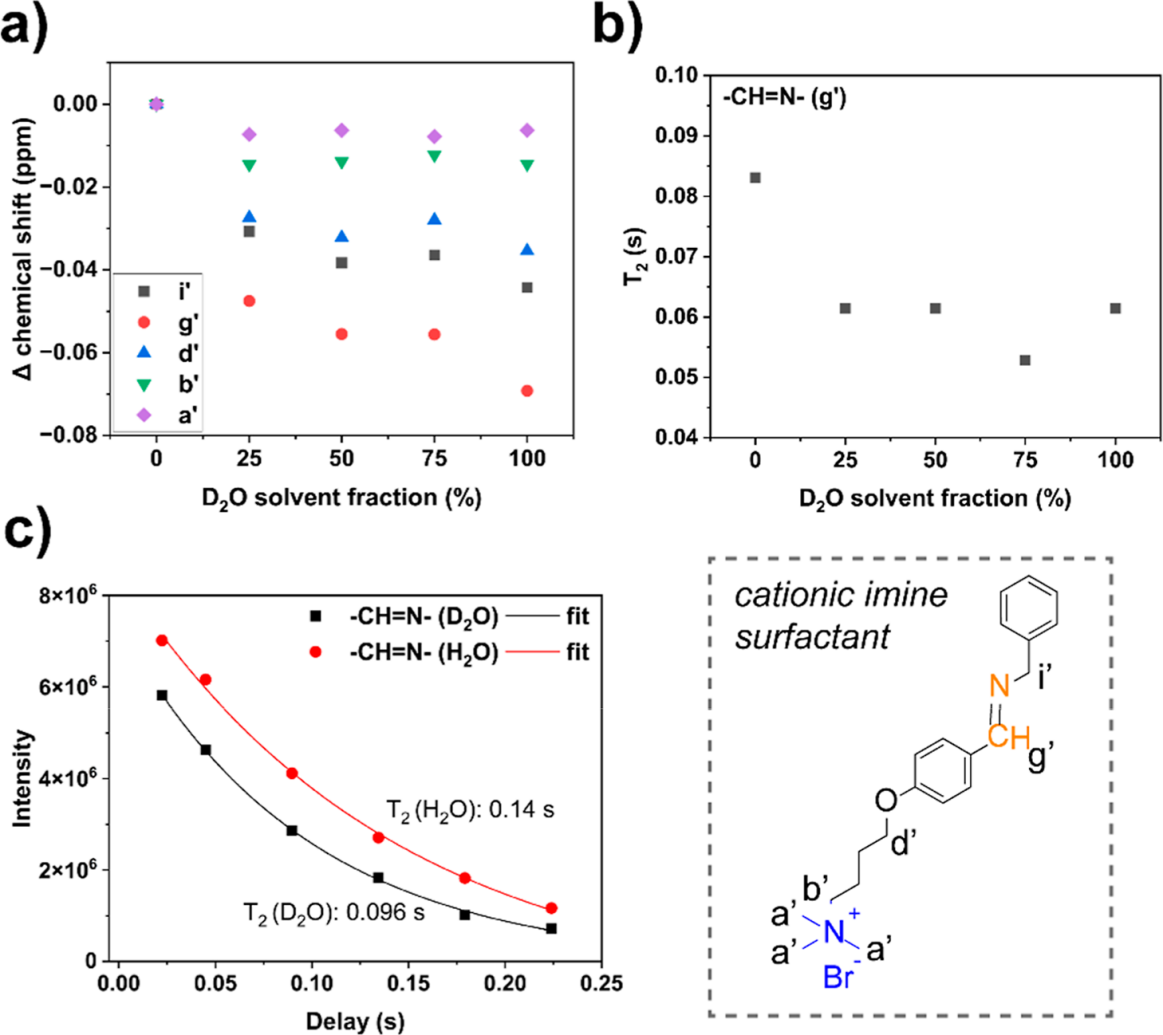
(a) Changes in proton chemical shifts of cationic imine surfactant from samples H-D 3.75-15-15 with varying D_2_O fractions, relative to those in nondeuterated buffer. The corresponding protons were marked on the chemical structure of imine surfactant shown with dashed box. Data were collected on a Bruker 600 MHz NMR spectrometer. (b) Transverse relaxation time (*T*_2_) of imine proton (–CH=N–, peak g’) from samples H-D 3.75-15-15 with increasing D_2_O fraction, measured on a Bruker 600 MHz NMR spectrometer. (c) Exponential decay fitting curve of imine motif (–CH=N–) from carbon transverse relaxation experiments at 850 MHz for samples H3.75-15-15 and D3.75-15-15.

Deuterium-dependent transverse relaxation times (*T*_2_) of equilibrated H-D 3.75-15-15 also provided insights into local motional dynamics for nucleus. If the interactions among imine assemblies were strongly enhanced by D_2_O, local motion would be restricted and *T*_2_ value would decrease. Then the T_2_ data were analyzed with inverse Laplace transform (ILT), which helped resolve different motional states of proton.^48,49^ We found that imine proton (g′) displayed a single, restricted motional state whose *T*_2_ decreased dramatically from 0.083 to 0.061 s, as D_2_O fraction rose from 0% to 100% (Figure 4b). This restricted local dynamics of imine proton (–CH=N–) propagated to the directly bonded carbon, which showed a shorter carbon *T*_2_ in D_2_O buffer (0.096 s) than in H_2_O buffer (0.14 s) (Figure 4c). The other two selected protons of imine surfactant displayed two motional populations (long *T*_2_ for free motion and short *T*_2_ for restricted motion), and their *T*_2_ change of restricted components was much smaller than that of imine proton when increasing D_2_O fraction (Figure S25). These observations indicate that the imine assemblies experience substantially more restricted local motion in the phase-separated system, consistent with strengthened intermolecular association in D_2_O. Control T_2_ measurement of imine on binary B-NH_2_/CS-CHO system displayed nearly unchanged value at 0.443 s across the full D_2_O range, except for a small decrease to 0.419 s at 75% D_2_O (Figure S24b). This modest decrease by 5% was substantially smaller than that of 26% observed in the phase-separated ternary system, supporting the conclusion that the dominant isotope effect emerged from LLPS-associated assembly rather than from the imine species alone.

To validate whether microenvironment within LLPS droplets was related to hydrophobic aggregation, turbidity tracking was carried out in D2.5-20-20 containing deuterated acetonitrile, a low-polarity solvent known to weaken hydrophobic interaction (Figure S26). At low cosolvent fractions (<1 vol %), LLPS kinetics were already strongly affected. Increasing the acetonitrile fraction from 0.2 to 0.5 vol % substantially prolonged the induction time and reduced the maximum turbidity, while the apparent imine yield remained comparable. At higher acetonitrile contents (≥1 vol %), LLPS was fully suppressed, likely because the cosolvent altered both the solvent properties and imine formation. The low-acetonitrile regime therefore provides supporting evidence that weakening hydrophobic association disfavors droplet formation.

Taken together, the solvent-isotope effect on LLPS appears to arise from multiple solvent-dependent contributions. D_2_O favors early association or nucleation of TAM–imine complexes and also strengthens hydrophobic association to facilitate LLPS; whereas, the amount of imine formed is only weakly affected by solvent isotope. These combined effects make phase separation more favorable in D_2_O than in H_2_O and provide a basis for spatiotemporal control of droplet formation. Notably, the accelerated LLPS observed in D_2_O cannot be explained by its higher viscosity. In a diffusion-controlled growth regime, droplet growth proceeds through diffusion of molecules from the supersaturated soluble phase into droplets.^50,51^ Thus, a higher viscosity would be expected to reduce diffusivity and slow growth rather than accelerate it.

We also noted that the labile amide N–H sites on TAM may undergo H/D exchange in D_2_O.^52^ Because N-D supports a slightly stronger and more stable hydrogen bond than N–H, such exchange may provide a secondary contribution to phase separation. Hydrogen-bond reinforcement is unlikely to be the dominant origin of the observed behavior, because hydrogen bonding requires stricter geometric and solvation conditions than the hydrophobic association implicated here.

### 2.5 H_2_O–D_2_O-Gradient Enabled Directional Movement of LLPS Droplets

Next we explored whether a solvent isotope composition gradient could drive directional transport of LLPS droplets via Marangoni flow, since D_2_O has a lower surface tension than H_2_O.^39^ Surface-tension measurements confirmed that the surface tension of buffer decreased from 69.44 to 62.94 mN/m as the D_2_O fraction increased from 0% to 100% and that the actual sample pairs H/D3.75-15-15 and H/D2.5-20-20 retained surface-tension differences of 4.1 and 6.7 mN/m, respectively (Figure 5a,b). These results indicate that a measurable surface tension gradient can be established under the working conditions.

**Figure 5:**
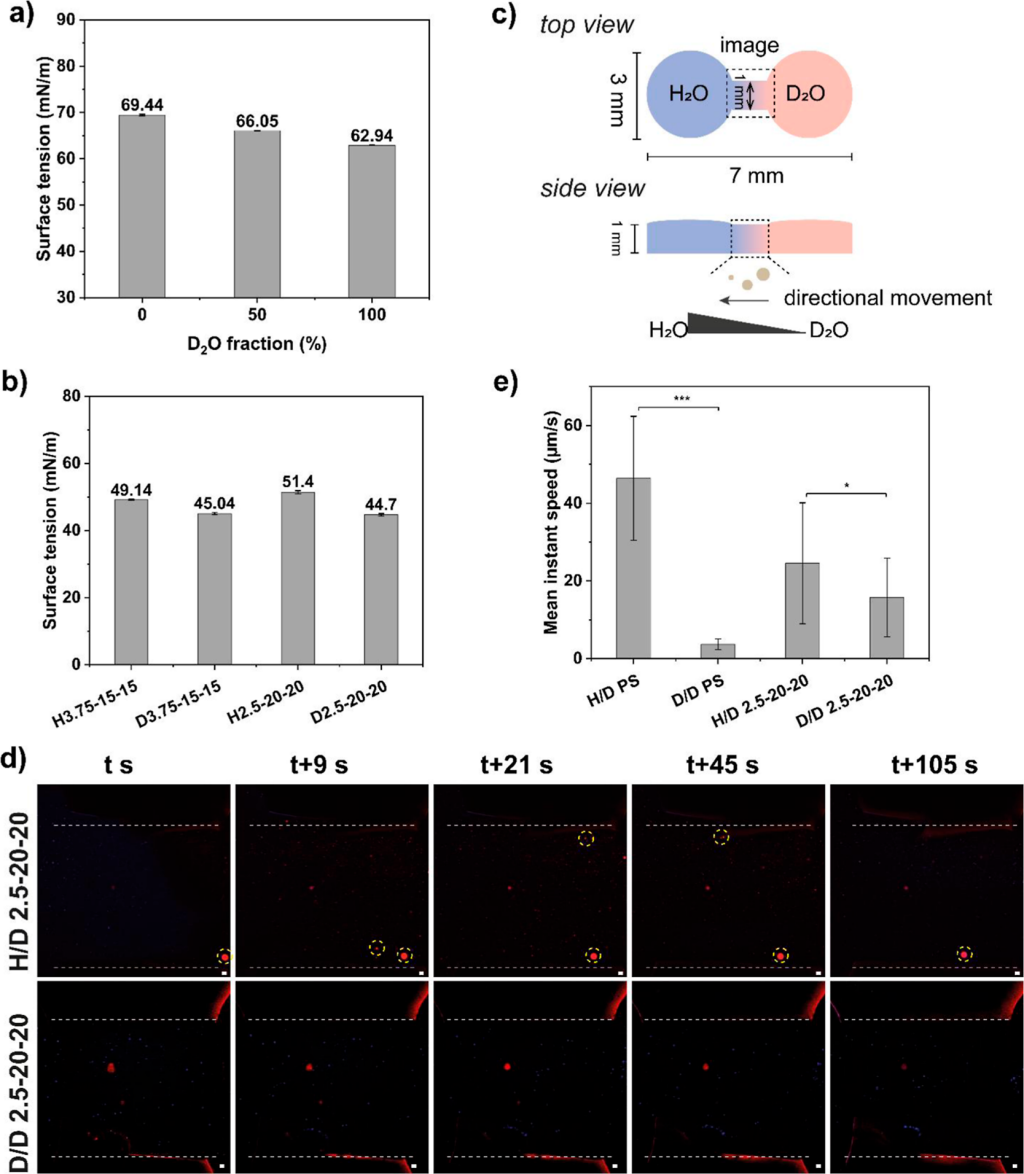
(a) Surface tensions of buffers containing 0%, 50%, and 100% D_2_O. (b) Surface tensions of H3.75-15-15, D3.75-15-15, H2.5-20-20, and D2.5-20-20. Surface tension was measured with pendant drop method by Theta Flex optical tensiometer. Data are shown as mean ± standard deviation (*n* = 3). (c) Schematic view of the custom-built two-well microscopy setup for imaging droplet movement. The left well (blue area) was filled with H_2_O or D_2_O samples stained with Methylene blue, while the right well (pink area) contained D_2_O samples stained with Nile red. (d) Snapshots from a time-lapse imaging session in the connecting tunnel where fluids from left and right side converged. H/D2.5-20-20 denoted that H2.5-20-20 and D2.5-20-20 were placed in the left and right well, respectively. D/D2.5-20-20 denoted that both wells were filled with D2.5-20-20. Dashed line indicated the tunnel boundaries, and dashed circle highlighted the moving droplets. Scale bar: 30 μm. (e) Mean instant speed of objects moving in time-lapse projects for H/D PS, D/D PS, H/D 2.5-20-20, and D/D 2.5-20-20. A total of 27–37 objects per condition were analyzed across three independent experiments. Data are shown as mean ± standard deviation. Statistical significance is evaluated by Mann–Whitney test (*p < 0.05, ***p < 0.001).

To impose the solvent isotope gradient and visualize the droplet movement, we used a connected two-well setup and imaged the junction where fluids from the left and right wells converged (Figure 5c). Hydrophilic dye methylene blue was added into the sample from the left side, and hydrophobic dye Nile red was applied to the sample from the right side. Both fluorescent dyes were used as model molecular cargoes and to track solvent and droplets. Upon fluids contacting in the central tunnel, a H_2_O–D_2_O solvent gradient could be formed immediately and expected to facilitate directional movement of LLPS droplets passing through the central tunnel, which was imaged under a laser confocal microscope.

To verify whether this gradient can drive LLPS droplets, we first tested H/D3.75-15-15, specifically loading LLPS-free sample (H3.75-15-15) into the left well and droplet-forming sample (D3.75-15-15) into the right well. The same component concentrations were used here to minimize the pitfalls driven by concentration. As expected, we found clear, directional migration of Nile red-stained droplets from the D_2_O side to the H_2_O side, consistent with motion along the initial solvent isotope gradient (Figure S27 and Movie S1). To substantiate the feasibility of solvent isotope design, we placed the same D3.75-15-15 in both wells (denoted as D/D3.75-15-15) and then imaged following the same procedures (Figure S27 and Movie S2). It turned out that after abolishing the isotopic gradient, directional transport of droplets was eliminated, indicating that the solvent isotope gradient was required.

For H/D3.75-15-15, one may be concerned that droplets occurring only in D_2_O side rendered these results insufficient to support proposed directed movement driven by solvent isotope gradient. We therefore prepared another set of paired samples H/D2.5-20-20 with a composition ratio that supported LLPS in both wells. It turned out that even with droplets present on both sides, droplets still moved significantly and predominantly from D_2_O side to H_2_O side, confirming that the solvent isotope gradient, rather than unequal phase behavior, drove this motion (Figure 5d and Movie S3). The moving Nile red-stained droplets were gradually costained by methylene blue, suggesting continuous exchange with the surrounding medium while moving along the gradient. For control-paired samples D/D2.5-20-20 lacking a gradient, droplets moved without persistent directional bias, either to the left or to the right within the narrow connecting channel (Figure 5d and Movie S4). Quantitative trajectory analysis showed that the mean instant speed in H/D2.5-20-20 was 24.55 ± 15.56 μm/s, corresponding to 2.67 ± 1.64-fold that of D/D2.5-20-20 (15.73 ± 10.10 μm/s), with statistical significance by the Mann–Whitney test (*p* < 0.05) (Figure 5e). Although gradients in the concentrations of free components may also contribute to the observed motion in sample pairs, an inert-particle control supports a major role for Marangoni-driven transport. Polystyrene particles, which do not undergo composition-dependent assembly, also moved much faster under the H/D condition (46.39 ± 15.97 μm/s) than under the D/D control, with high statistical significance (*p* < 0.001) (Figure 5e). This result strongly supports a transport mechanism dominated by the H_2_O–D_2_O-induced surface-tension gradient. Therefore, the proposed H_2_O–D_2_O isotopic gradient is sufficient to trigger droplet motion from D_2_O-rich to H_2_O-rich regions, simultaneously enabling the substance exchange with the surroundings.

## 3 Conclusion

We establish an approach of isotopic-solvent control, specifically deuterated water (D_2_O) versus light water (H_2_O), to integrate localized formation and directed transport within an aqueous synthetic system. The designed reactive small-molecular system involves dynamic imine chemistry and is composed of anionic macrocycles TAM, aldehyde-modified cationic surfactant CS-CHO, and benzylamine B-NH_2_. The in situ-generated cationic imine surfactant complexes with TAM to enable LLPS, with faster and more pronounced droplet formation observed in D_2_O buffer than H_2_O buffer. The isotope effect does not primarily arise from changes in imine yield. Instead, D_2_O favors early TAM–imine association and strengthens hydrophobic association among imine assemblies. This conclusion is supported by larger enthalpy change, pronounced proton chemical shifts changes, and shorter transverse relaxation times, all consistent with D_2_O-amplified noncovalent interactions.

In addition to D_2_O-regulated droplet formation, an imposed H_2_O–D_2_O isotopic gradient establishes a surface tension difference that drives gradient-guided passive transport of droplets from D_2_O-rich toward H_2_O-rich regions. These droplets can move faster under isotope gradient control than those without a gradient. During movement, the abilities of LLPS droplets for transporting and exchanging are demonstrated using fluorescent dyes as model cargos. Therefore, by harnessing isotopic solvent control, this work demonstrates that localized growth and directional movement could be achieved within a fully aqueous synthetic system, providing insights into engineering more advanced and complex lifelike synthetic systems. Preliminary scope studies further indicate that this solvent-isotope effect is system-dependent (Table S1 and Figure S28). Using structurally related macrocycles can display different or even opposite behavior. D_2_O-promoted phase separation therefore depends sensitively on the balance between electrostatic and hydrophobic interactions in a given molecular system.

## Supplementary Material











## Notes

Experimental procedures; turbidity tests; NMR data; and supplementary figures (PDF)
Confocal microscopy recording of Marangoni flow in H/D3.75-15-15 (WMV)
Confocal microscopy recording of steady convection in D/D3.75-15-15 (WMV)
Confocal microscopy recording of Marangoni flow in H/D2.5-20-20 (WMV)
Confocal microscopy recording of steady convection in D/D2.5-20-20 (WMV)
