# Supplementary material for: Deuterated Water Accelerates Phase-Separated Droplet Formation and Enables Directional Motion
Source: J Am Chem Soc. 2026 Jun 9;148(24):24900–10. doi: 10.1021/jacs.6c03395 (PMC13307370; doi:10.1021/jacs.6c03395)
Supplement: Supplementary file 1 [file ja6c03395_si_001.pdf]

Supplementary information

Deuterated Water Accelerates Phase-Separated Droplet Formation and Enables Directional Motion

Caihong Lin [1], Jingjing Yu [1‡], Dawei Qi [1,2‡], Xuncheng Shi [1], Tuomas Niemi-Aro [3], Jianwei Li [4]*

1 MediCity Research Laboratory, University of Turku, Tykistökatu 6, 20520 Turku (Finland)

2 NMR Research Unit, Faculty of Science, University of Oulu, Pentti Kaiteran katu 1, 90014 Oulu (Finland)

3 Institute of Biotechnology, Helsinki Institute of Life Science, University of Helsinki, Viikinkaari 1, 00014 Helsinki (Finland)

4 Macao Institute of Materials Science and Engineering (MIMSE), Faculty of Innovation Engineering, Macau University of Science and Technology, Taipa, 999078 Macao (China)

‡ Jingjing Yu and Dawei Qi contributed equally to this work.

* Corresponding author, email: lijianwei@must.edu.mo

Table of Content

1. Experimental procedures 1

1.1 Materials 1

1.2 Synthetic protocols of compounds 1

1.2.1 Synthesis of TAM 1

1.2.2 Synthesis of CS-CHO 3

1.3 Ultraviolet visible (UV-vis) spectroscopy 5

1.4 Dynamic light scattering (DLS) and Zeta potential measurement 5

1.5 Nuclear magnetic resonance (NMR) spectroscopy 5

1.5.1 Proton 6

1.5.2 Diffusion ordered spectroscopy (DOSY) 6

1.5.3 Nuclear Overhauser Effect spectroscopy (NOESY) 6

1.5.4 Relaxation 6

1.6 Determination of produced imine by UPLC-MS 6

1.7 Isothermal titration calorimetry (ITC) 8

1.8 Confocal fluorescence microscopy 8

1.8.1 Sample preparation 8

1.8.2 Confocal microscopy about droplet mobility 8

1.8.3 Fluorescence recovery after photobleaching (FRAP) 8

2. Supplementary Figures 9

3. References 21

1. Experimental procedures

1.1 Materials

All solvents were purchased in analytical grade from Thermo Fischer Scientific and used without further purification. 4,4'-Biphenyldicarboxylic acid and 4-hydroxybenzaldehyde were obtained from BLDpharm. N-Hydroxy succinimide (NHS), 4-dimethylaminopyridine (DMAP), L-cysteine, and 1,4-dibromobutane, benzylamine (B-NH_2) were obtained from Tokyo Chemical Industry (TCI). 1-Ethyl-3-(3-dimethylaminopropyl)carbodiimide (EDCI) was sourced from abcr GmbH. Sodium hydroxide (NaOH) and hydrochloric acid (HCl) were purchased from Sigma Aldrich. Deuterated solvent were acquired from Eurisotop and used as received. Milli-Q (MQ) water was produced using Milli-Q EQ 7000 system (Merck Millipore).

Buffer solution was prepared using 160 mM NaH_2PO_4, and adjusted to pH 11.4 by adding NaOH, as measured using a Mettler Toledo pH meter. The pH meter was calibrated using standard buffer solutions prior to use. Deuterated phosphate buffer was prepared by lyophilizing non-deuterated buffer and reconstituting it with an equal volume of deuterated water. The final pD was calculated using the formula: pD = pH + 0.4.[1] The stock concentrations of TAM, CS-CHO, B-NH_2 were 20, 40, and 80 mM, respectively, unless stated otherwise. Samples were prepared by mixing appropriate volumes of TAM, B-NH_2 and buffer, followed by the addition of CS-CHO in a glass vial. The final solution volume was typically 600 μL. Samples in gradient deuterated buffer were prepared by freeze-drying pre-mixed samples ([TAM]:[CS-CHO]:[B-NH_2] = 3.75 mM: 15 mM: 15 mM) in fully non-deuterated buffer, followed by reconstitution with corresponding volumes of deuterated water and equilibration for at least 8 hours.

1.2 Synthetic protocols of compounds

1.2.1 Synthesis of TAM

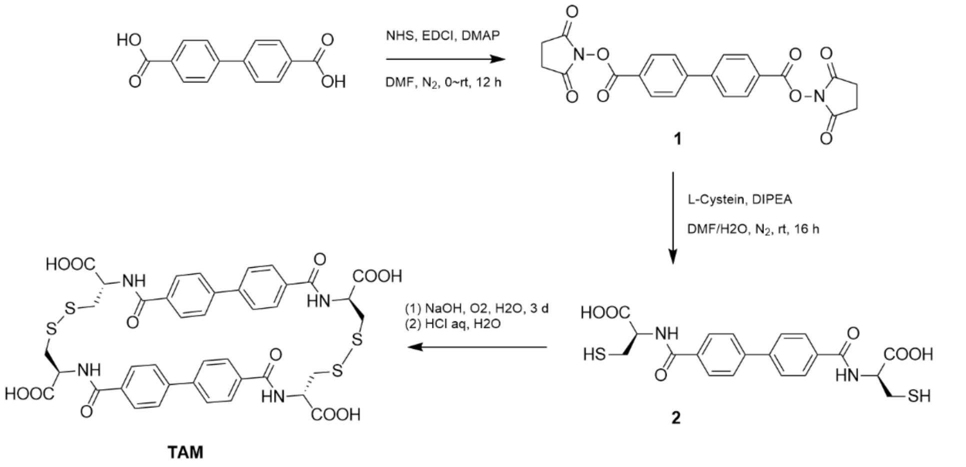

Scheme S1. The synthetic route of cyclic dimer **TAM**.

The synthesis was performed according to a modified literature procedure.[2] 4,4'-Biphenyldicarboxylic acid (10 g, 41.3 mmol) was dissolved in 100 mL of N,N-dimethylformamide (DMF), and cooled in an ice bath. N-hydroxy succinimide (NHS) (11.4 g, 99.1 mmol) was added, followed by catalytic 4-dimethylaminopyridine (DMAP) and 1-ethyl-3-(3-dimethylaminopropyl)carbodiimide (EDCI) (19 g, 98.9 mmol). The reaction mixture was then heated to 60 °C and stirred for 12 hours under a nitrogen atmosphere. After cooling to room temperature, the reaction mixture was poured into water for precipitation. The precipitate was collected by filtration through a Buchner funnel and

washed three times with water, yielding intermediate product **1** as white solid (15.6 g, 87% yield) without further purification.

Compound **1** (4.3 g, 9.8 mmol) was dissolved in 180 mL DMF, and mixed with an aqueous solution of L-cysteine (3 g, 24.8 mmol, in 20 mL H_2O). N,N-diisopropylethylamine (DIPEA) (6.9 mL, 39.7 mol) was added, and the mixture was stirred at room temperature for 16 hours. Reaction progress was monitored by thin layer chromatography (TLC) using a 10:1 mixture of dichloromethane and methanol as the eluent. The reaction mixture was then concentrated under reduced pressure and the resulting crude product was poured into water to precipitate the solid. The precipitate was collected by filtration and redissolved in NaOH solution (3.9 g, 98 mmol). The solution was bubbled with air for three days to facilitate oxidation. Afterward, the solution pH was adjusted to acidic (~ 2) with 1 M HCl to induce precipitation. The resulting solid was filtered, washed three times with water, and dried by freeze-drying to yield TAM as white powder (2.7 g, 62% yield). The chemical structure of TAM was identified by ^{1}H NMR spectroscopy.

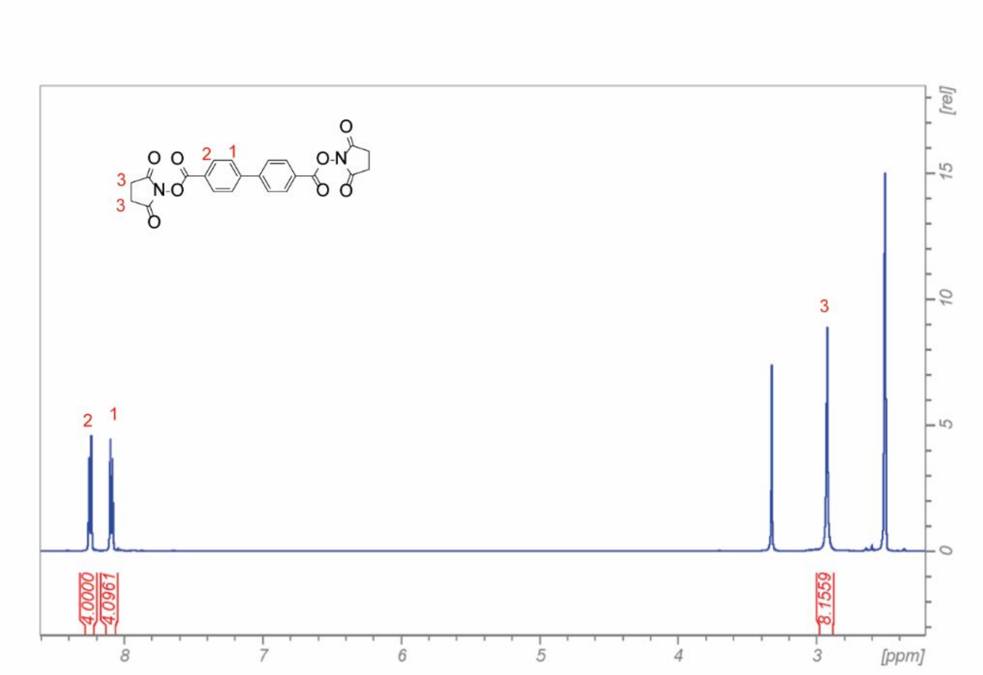

Figure S1. 1D proton spectra of esterification product **1** (DMSO-d_6, 500 MHz, 298 K).

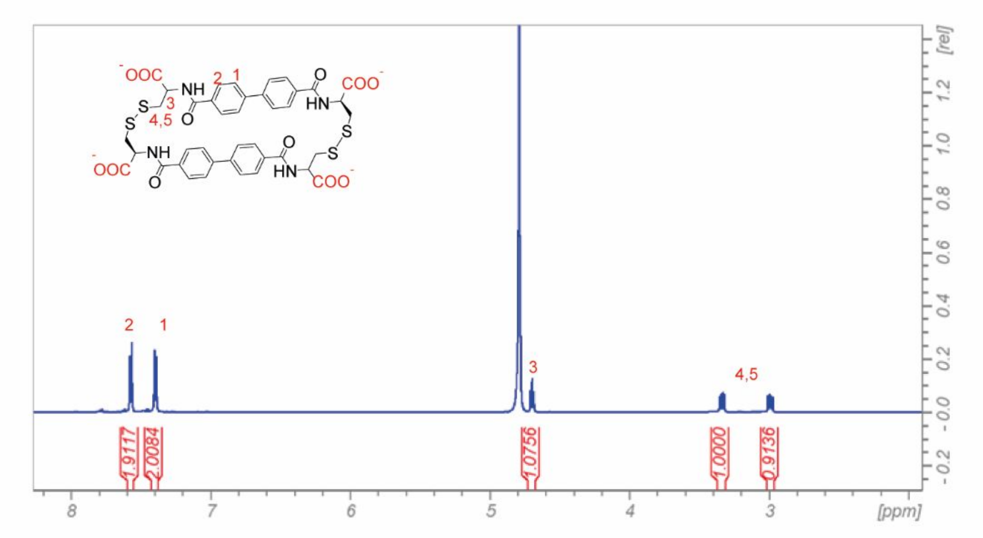

Figure S2. 1D proton spectra of cyclic dimer **TAM** (D_2O buffer, pD 11.4, 600 MHz, 298 K).

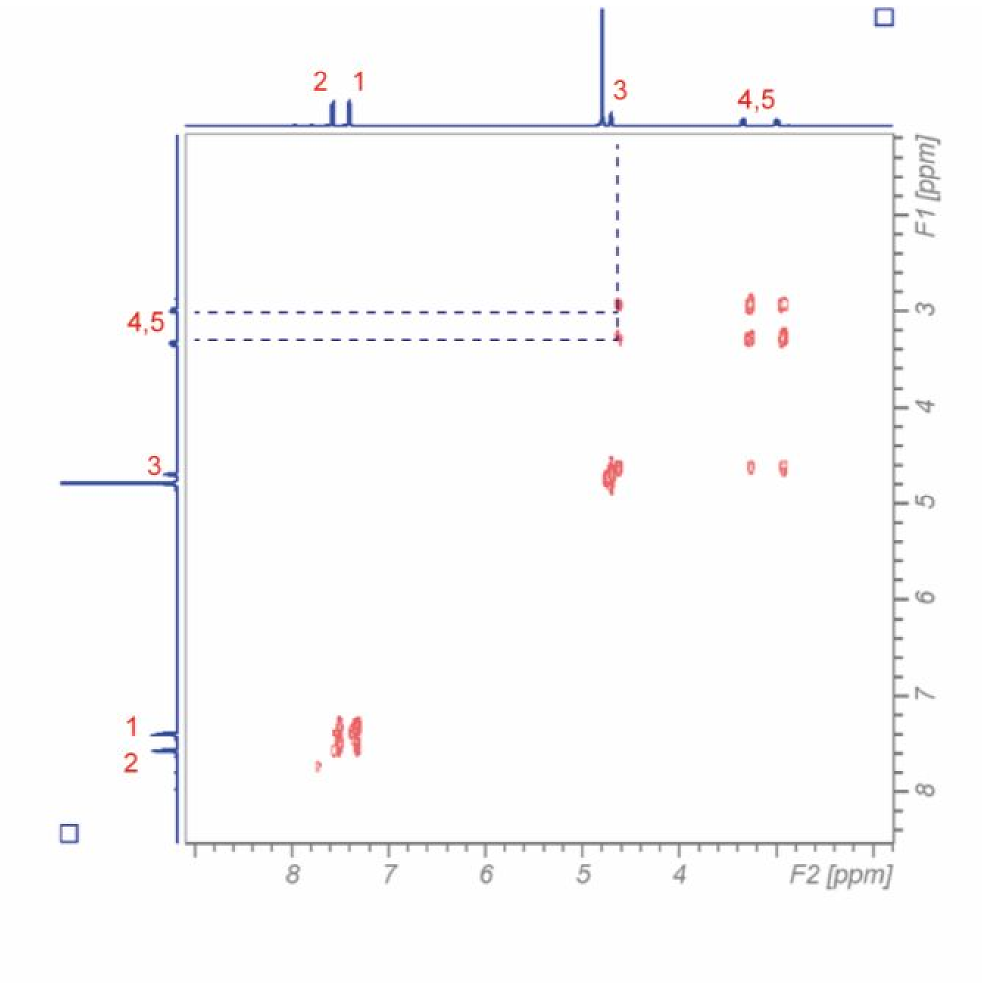

Figure S3. 2D ^{1}H-^{1}H COSY spectra of cyclic dimer **TAM** (D_2O buffer, pD 11.4, 600 MHz, 298 K).

1.2.2 Synthesis of CS-CHO

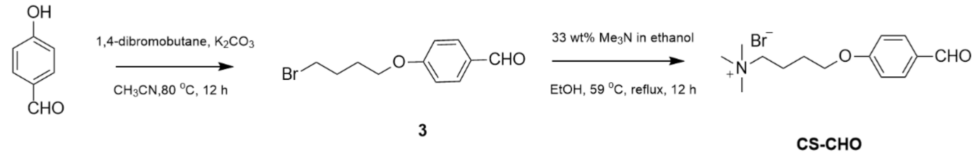

Scheme S2. The synthetic route of cationic surfactant CS-CHO.

4-Hydroxybenzaldehyde (5 g, 40.9 mmol) was dissolved in 140 mL acetonitrile (CH_3CN), followed by the addition of 1,4-dibromobutane (43.8 g, 202.9 mmol). Dry potassium carbonate (17 g, 123.2 mmol) was then added, and the reaction mixture was refluxed at 80 °C for 12 hours. After completion, the mixture was filtered to remove inorganic salt, and the filtrate was concentrated under reduced pressure. The resulting crude oil was purified by flash column chromatography using petroleum ether / ethyl acetate mixture (4 : 1, v/v) as the eluent. The product was dried using a rotary evaporator to afford intermediate product **3** as clear oil (4.8 g, 46% yield).

The bromine-substituted intermediate **3** (1 g, 3.9 mmol) was dissolved in 10 mL ethanol. Trimethylamine in ethanol (33 wt%, 14 mL) was then added, and the reaction mixture was refluxed at 59 °C for 12 hours. The targeted product CS-CHO was obtained as white solid (0.9 g, 75% yield) by rotary evaporation. The chemical structure of CS-CHO was confirmed using ^{1}H NMR spectroscopy.

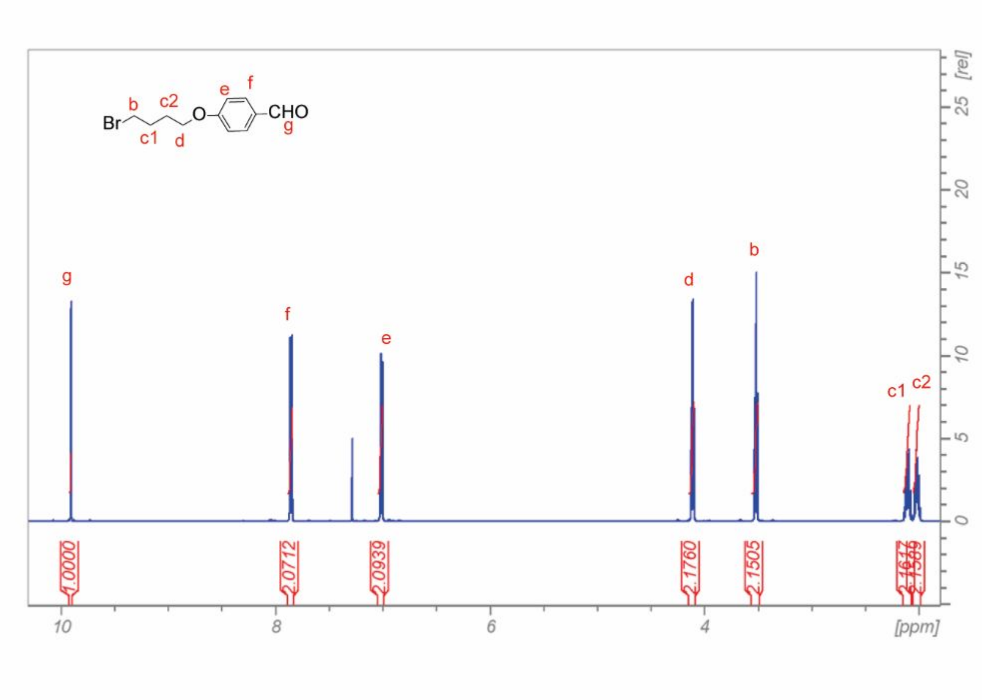

Figure S4. 1D proton spectra of bromine-substituted product **3** (CD_3Cl, 500 MHz, 298 K).

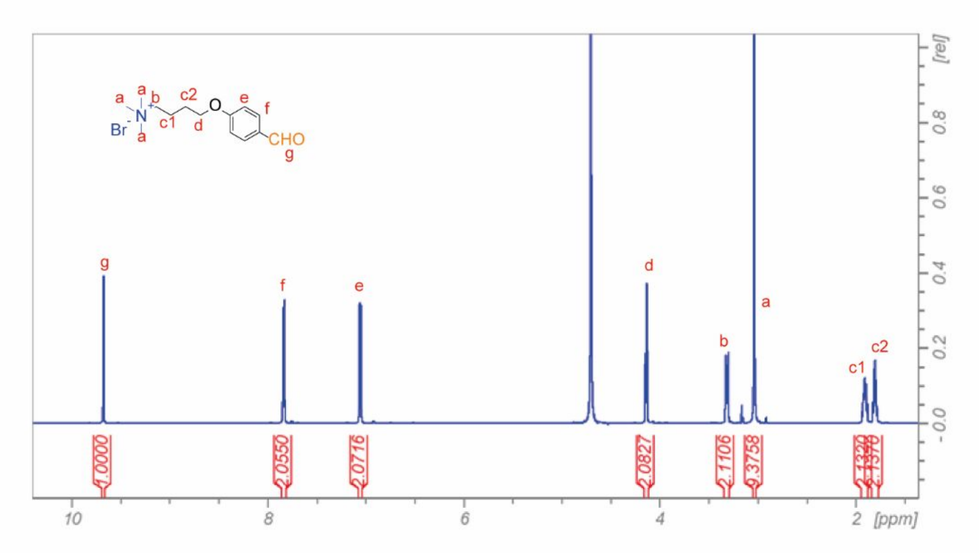

Figure S5. 1D proton spectra of cationic surfactant **CS-CHO** (20 mM, D_2O buffer, pD 11.4, 600 MHz, 298 K).

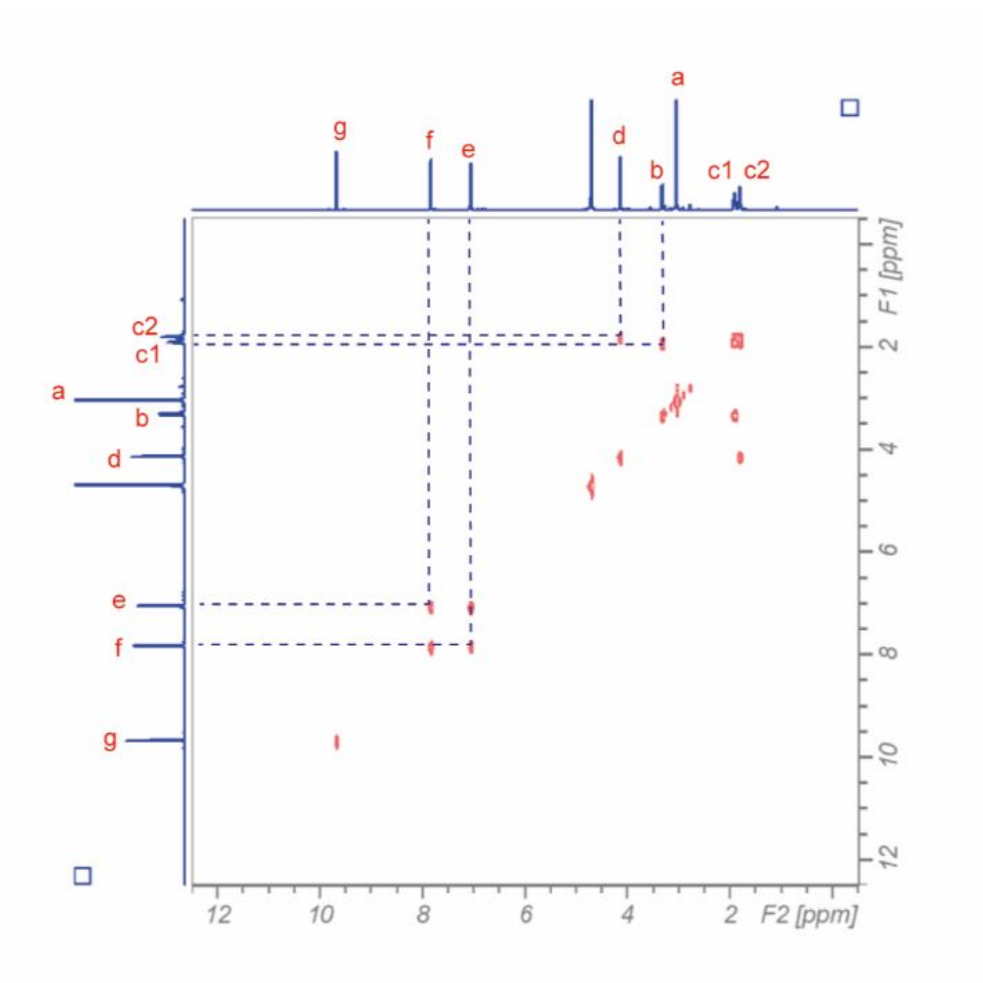

Figure S6. 2D ^{1}H-^{1}H COSY spectra of cationic surfactant **CS-CHO** (20 mM, D_2O buffer, pD 11.4, 600 MHz, 298 K).

1.3 Ultraviolet visible (UV-vis) spectroscopy

UV-vis measurements were conducted on a PerkinElmer Lambda 365 UV/Vis spectrometer equipped with Peltier temperature control. Samples were freshly prepared by mixing the appropriate amounts of stock solutions and buffer in a microcentrifuge tube. After vigorous vortex, sample was quickly transferred to a quartz cuvette (light path: 5 mm). For time-dependent measurements, spectral scans between 190 nm and 700 nm was collected at 10-minute intervals for 8 hours. All measurements were performed at 25 °C with gentle stirring. Turbidity curves were plotted by recording absorbance at a wavelength of 550 nm over time.

1.4 Dynamic light scattering (DLS) and Zeta potential measurement

DLS and Zeta potential measurements were carried out on Malvern Zetasizer Nano-ZS equipped with a He-Ne ion laser (633 nm), using disposable folded capillary cells (DTS1070, Malvern, UK). Samples D3.75-15-15 and H3.75-15-15 were gently vortexed and used without further dilution. Each measurement was conducted at 25 °C and repeated five times.

1.5 Nuclear magnetic resonance (NMR) spectroscopy

For samples prepared in non-fully deuterated buffers, a coaxial capillary filled with D_2O was placed inside the NMR tube for field-frequency lock. Trace amounts of acetonitrile (CH_3CN) was added in specific cases for internal

calibration of chemical shifts. Excitation sculpting method was used for water suppression. All measurements were performed at 25 °C, with chemical shifts expressed in parts per million (ppm). Data processing and fitting were performed using the Dynamic Center module in TopSpin 4.3.0 software.

1.5.1 Proton

Kinetic proton experiments were conducted at a 500 MHz Bruker AVANCE-Ⅲ NMR system with Cryo Probe, using zg30 Bruker pause program for samples prepared in fully deuterated buffer. Data was collected at various time points to observe reaction kinetics. For samples with varying D_2O fractions, 1D proton experiments were conducted at a Bruker 600 MHz AVANCE III NMR spectrometer equipped with a 5 mm double-resonance (X, 1H) broadband probehead (SmartProbe), using zgesgp Bruker pause program.

1.5.2 Diffusion ordered spectroscopy (DOSY)

DOSY experiments were conducted at a Bruker 850 MHz Avance III HD spectrometer equipped with cryogenically cooled 5 mm triple-resonance (H, C, N) probehead with Z-axis gradients, with maximum amplitude of 50 G/cm, using Bruker pulsed-field gradient pulse program stebpesgp1s for H_2O sample and ledbpgp2s for D_2O sample. Diffusion time interval (d20) was set at 100 ms and the gradient pulse strength (p30) at 1000 ms. The DOSYs measurements were recorded over 8 scans with a pulse gradient field strength varying from 2% to 98% in 128 steps. Translation diffusion coefficients (D) were determined from the signal decay in a series of spectra using the fitting function of vargrad.

1.5.3 Nuclear Overhauser Effect spectroscopy (NOESY)

^1H-^1H NOESY experiments were recorded at a Bruker 850 MHz Avance III HD spectrometer equipped with cryogenically cooled 5 mm triple-resonance (H, C, N) probehead with Z-axis gradients, with maximum amplitude of 50 G/cm, using the Bruker pause program noesyesgpph with number scan of 16, dummy scan of 32 and mixing time of 0.3 s. Cross peaks of a NOESY spectrum indicate which protons are close to each other in space.

1.5.4 Relaxation

Relation experiments were conducted at a Bruker 600 MHz AVANCE III NMR spectrometer equipped with a 5 mm double-resonance (X, 1H) broadband probehead (SmartProbe). Transverse relaxation times (T_2) were measured using Bruker Carr-Purcell-Meiboom-Gill sequence (cpmg_esgp2d) with 64 time points. Recovery times (t) ranged from 2 ms to 16 s. The resulting data was analyzed by inverse Laplace transform (ILT) to resolve components with different motional states and obtain their distributions.[3, 4]

^1H-^{13}C HSQC T_2 relaxation experiments were recorded at a Bruker 850 MHz Avance III HD spectrometer equipped with cryogenically cooled 5 mm triple-resonance (H, C, N) probehead with Z-axis gradients, with maximum amplitude of 50 G/cm, using the Bruker pause program hsqct2etgpsi3d.2. A series of CPMG loop counter (8 values, ranging from 1 to 10) were set, with a duration of 0.0224 s per loop. To prevent heating artifacts, loops were randomized. Each spectrum was acquired with 64 scans, and two-dimensional planes were acquired as an arrayed-experiment with 128 dummy-scans. Peak intensities were extracted and fitted to determine ^{13}C-T_2 value.

1.6 Determination of produced imine by UPLC-MS

To compare with NMR result, ultra-performance liquid chromatography-mass spectrometry (UPLC-MS) analysis by

quenching the equilibrium with $NaBH_4$ was chosen as an alternative technique.[5] 50 μL of sample at each time point was taken out to a microcentrifuge tube, followed by the addition of 5 equivalent of $NaBH_4$ and vortex, which reduced aldehyde group and imine to their corresponding non-dynamic alcohols and amines (Figure S7). After reaction for 10 minutes, these samples were diluted by 200 times in methanol and analyzed by UPLC-MS using a H_2O/CH_3CN gradient containing 0.1% TFA. The peaks of the corresponding reduction products in the chromatogram were identified with mass spectrometry (Waters Acquity RDa detector). The calibration curve for CS-CHO reduction product was obtained from subjecting samples with known quantities to UPLC analysis (Figure S8). The produced imine at different time point (t) was then determined according to the following formula: $[\text{imine}]_t = [\text{CS-CHO}]_0 - [\text{CS-CHO}]_t$, where $[\text{CS-CHO}]_0$ represented the initial concentration of CS-CHO.

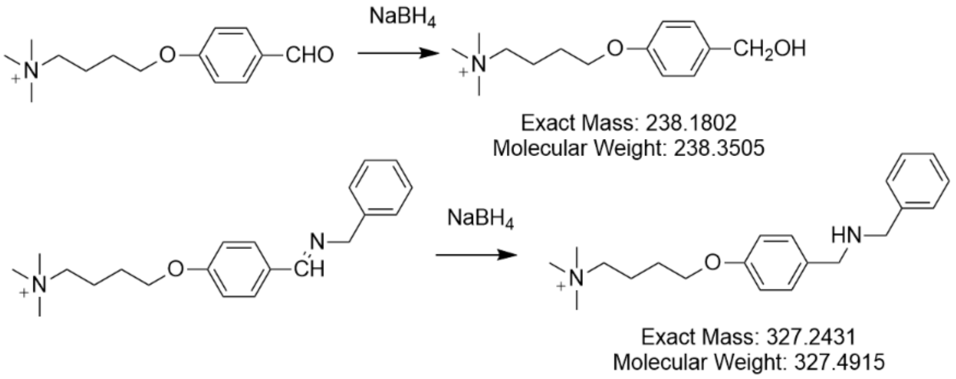

Figure S7. Chemical structures of non-dynamic alcohol and amine after $NaBH_4$ reduction in this multi-component system.

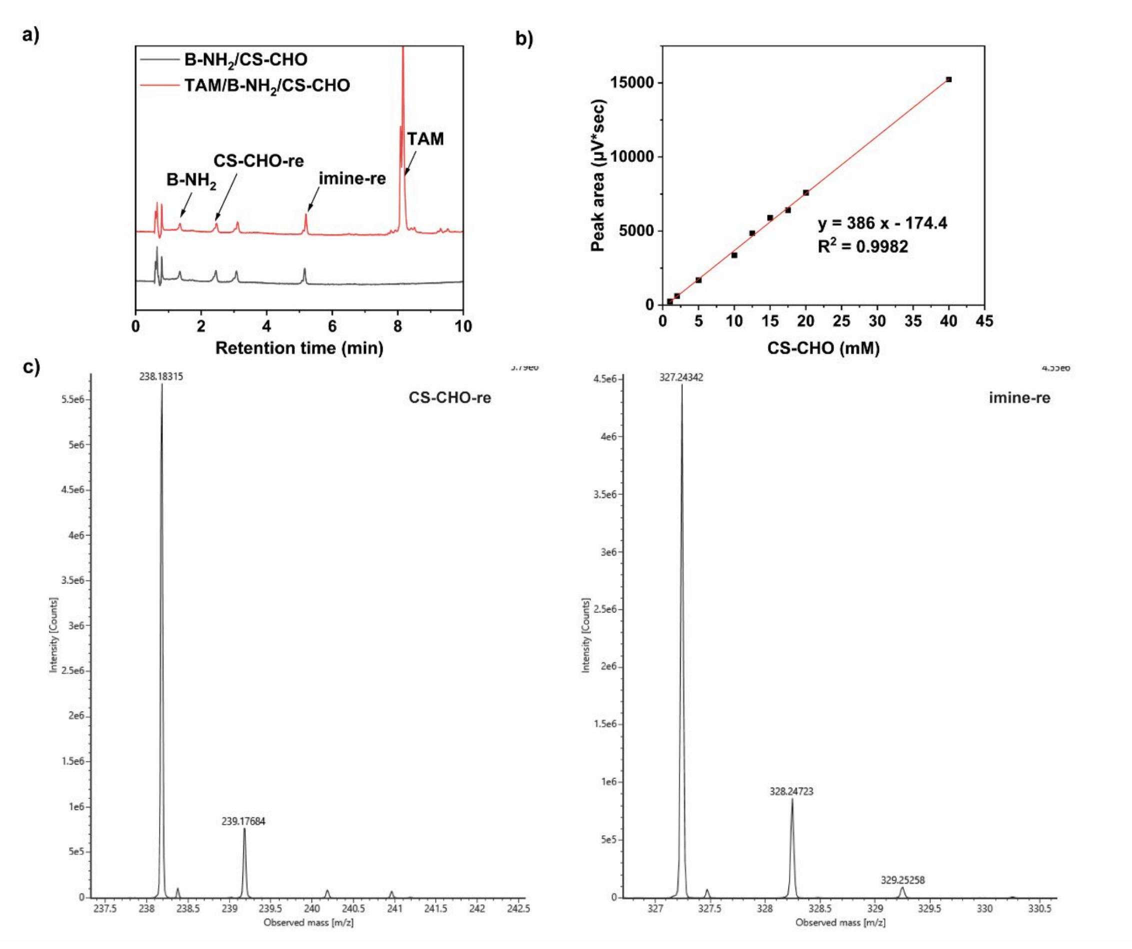

Figure S8. a) UPLC curves of samples B-NH_2/CS-CHO and TAM/B-NH_2/CS-CHO after reduction by $NaBH_4$. b) Calibration curve obtained by UPLC analysis of CS-CHO reduction product. c) Mass spectra for identification of peaks corresponding to reduction product in UPLC.

1.7 Isothermal titration calorimetry (ITC)

ITC experiments were to compare the released heat of a multicomponent system in deuterated buffer and nondeuterated buffer, and performed using MicroCal ITC200 (Malvern Panalytical). Each titration experiment involved the initial injection (0.5 μL), the second injection (1 μL) and the subsequent 18 injections (2 μL) of 30 mM TAM solution (either in D_2O buffer or H_2O buffer) with 5 min intervals into the sample cell containing the complex of 15 mM CS-CHO and 15 mM B-NH_2 (pre-equilibrium overnight, either in D_2O buffer or H_2O buffer). Both solutions in syringe and cell had the same pH of 11.4. The reference cell was filled with MQ water, and the experimental temperature and stirring rate were adjusted to 25 °C and 750 rpm, respectively. The heat of dilution, which was obtained by injection of TAM solution into corresponding buffer, was used as control. The final enthalpic curves reflecting the interactions between TAM and imine were generated by subtracting control heat from the titration data.

1.8 Confocal fluorescence microscopy

1.8.1 Sample preparation

For visualization, 2.5 μM Nile red (from 0.78 mM stock solution in MeOH) was added to a new glass vial, and waited for solvent vaporization, followed by the addition of freshly prepared samples and at least 4-hour equilibration. In order to differentiate droplets in the tests, a hydrophilic fluorescent dye methylene blue (36 μM, from 3.6 mM stock solution in MQ water) was used by directly adding to freshly prepared samples and equilibrating for 4 hours. Inert fluorescent polystyrene particles with an average diameter of 4 μm (from microParticles GmbH) is used as control and diluted 200-fold with D_2O or H_2O before use.

1.8.2 Confocal microscopy about droplet mobility

Confocal microscopy was conducted on a 3i CSU-W1 spinning disk microscope, utilizing 10x objective. A custom-built microscopy setup was composed of PVA-coated glass slide and 14-well self-adhesive Refeyn cassettes where 1 mm wide channel connected neighboring wells for purpose. The coating procedure followed previous reports to prevent wetting behavior.[6] Before imaging, the sample was vortexed gently, took 5 μL Nile red stained sample to the right well, and then took 5 μL methylene blue stained sample to the left well. To record the droplet dynamics process, every 3 seconds an image was collected for 5 minutes with laser λ_{ex} = 561 nm for nile red excitation and λ_{ex} = 640 nm for methylene blue excitation. The exposure time was set to 100 ms for both channels. The final image was collected using montage mode. Confocal images were processed in Slidebook 6 software and analyzed through Image J software (Fiji ImageJ>Plugins>Tracking>Manual Tracking).

1.8.3 Fluorescence recovery after photobleaching (FRAP)

FRAP experiment was conducted on a 3i CSU-W1 spinning disk microscope, utilizing 63x oil objective. FRAP was measure within a region of interest with a diameter of 10 pixels. The region of interest was subjected to 100%

beaching power of the λ_{ex} = 561 nm laser for 10 ms. Then, every 100 ms an image was recorded for 20 s. Three FRAP experiments were conducted for each condition and averaged. Image analysis for drift correction and quantification of fluorescent intensity was performed in Fiji ImageJ, The fluorescent intensity was normalized with the following equation:

$$F(t) = \frac{(T_0 - B_0)(I_t - B_t)}{(T_t - B_t)(I_0 - B_0)}$$

Here F(t) is the normalized fluorescent intensity at time t. I(t) represents the fluorescence intensity measured within the bleached region of interest (ROI) at time t, T(t) represents fluorescence intensity measured within an unbleached reference droplet at time t and B(t) represents background intensity at time t.

2. Supplementary Figures

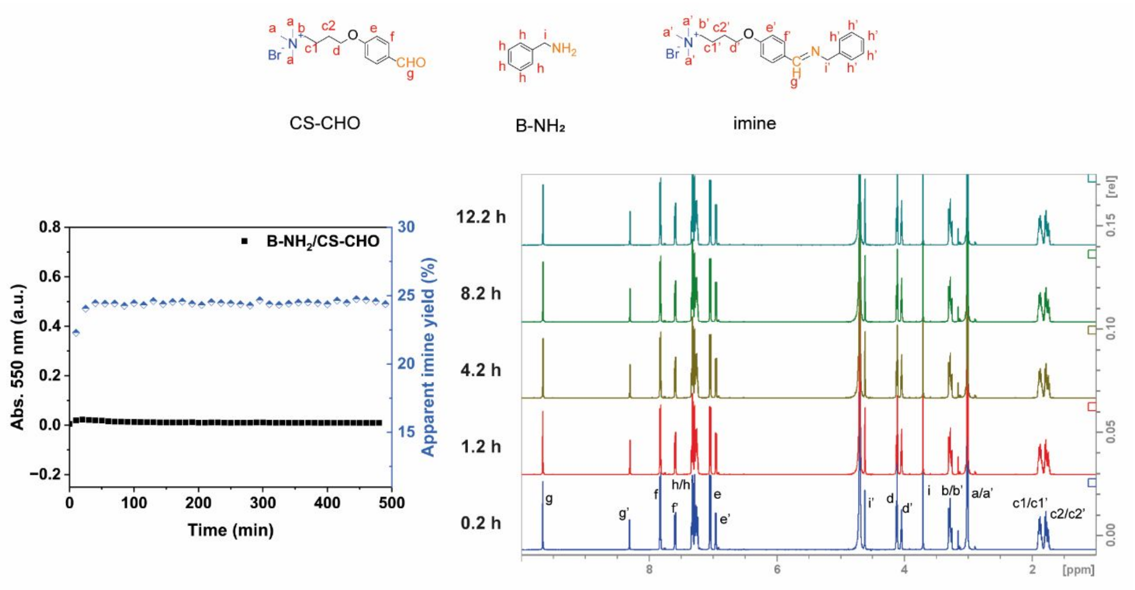

Figure S9. Turbidity test and apparent imine yield of sample B-NH_2/CS-CHO with a concentration ratio of 20 mM : 20 mM in D_2O buffer (pD 11.4). The apparent imine yield rapidly reached a maximum of 24.4%, and then remained stable. Right panel shown time-dependent proton NMR spectra (500 MHz) of same sample which displayed negligible chemical shift changes.

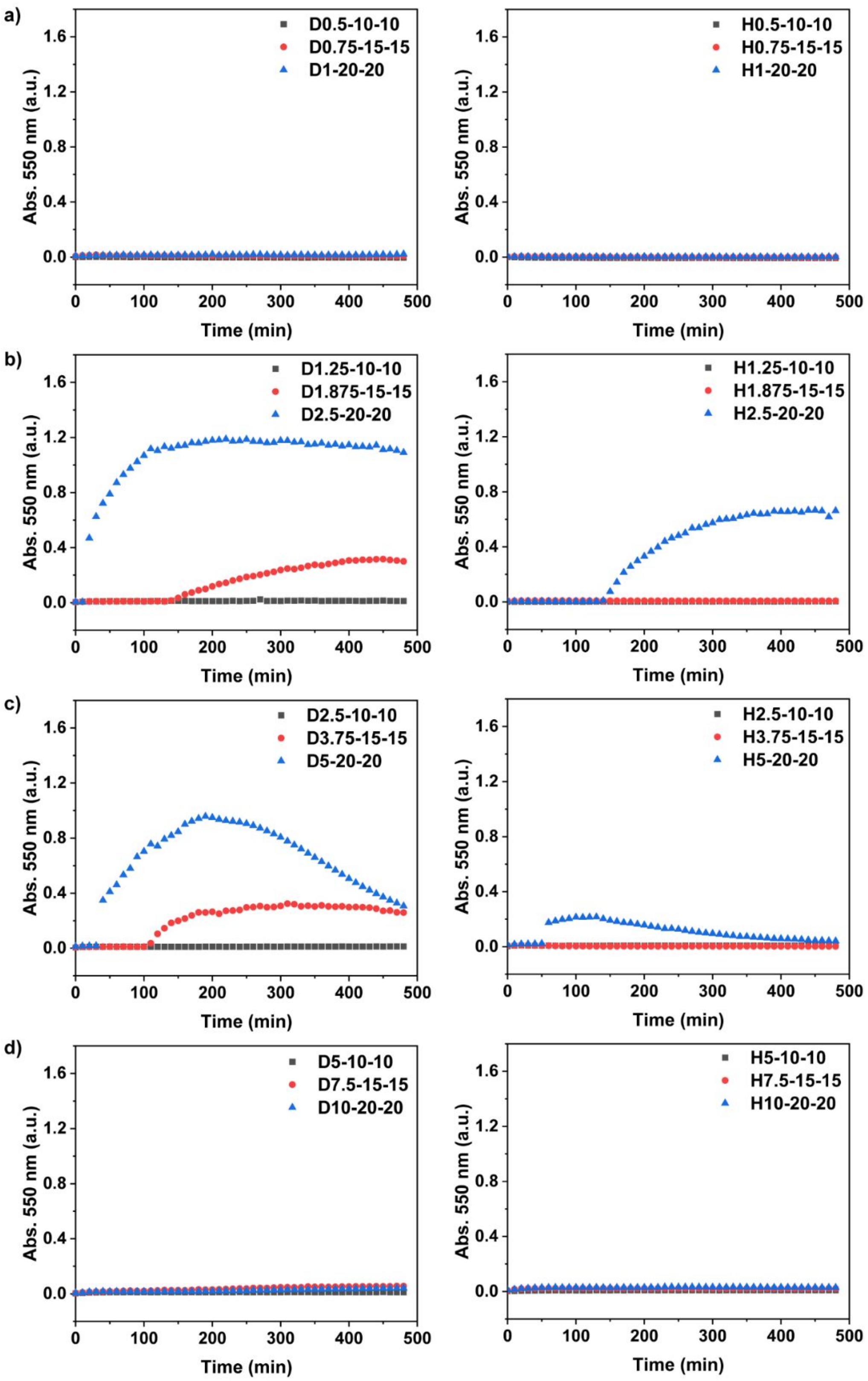

Figure S10. Turbidity measurements of the TAM/CS-CHO/B-NH_2 system at varying concentration ratios in D_2O (left panel) and H_2O (right panel) phosphate buffers, respectively. The stoichiometric ratios of TAM:CS-CHO:B-NH_2 were a) 1:20:20, b) 1:8:8, c) 1:4:4, d) 1:2:2. Absorbance change at 550 nm (A550) was recorded using UV-Vis spectrometry for each sample.

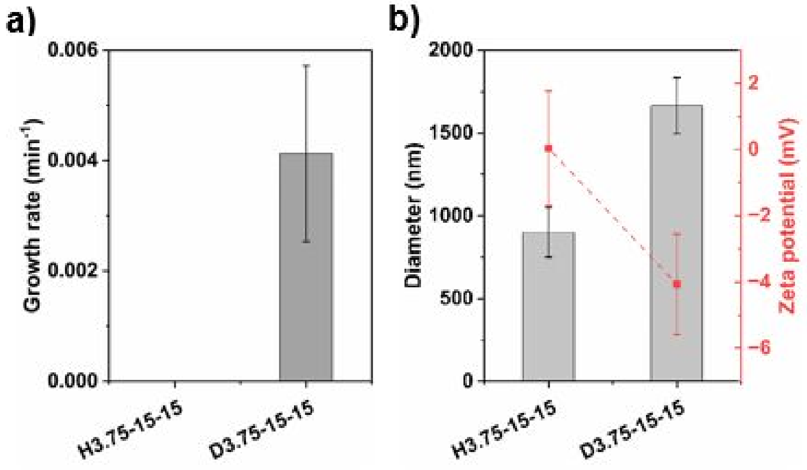

Figure S11. a) Comparison of growth rate, b) average droplet diameter and Zeta potential of samples D3.75-15-15 and H3.75-15-15 DLS analysis and Zeta potential measurements are completed on Zetasizer Nano ZS. Data are shown as mean ± standard deviation (n = 3).

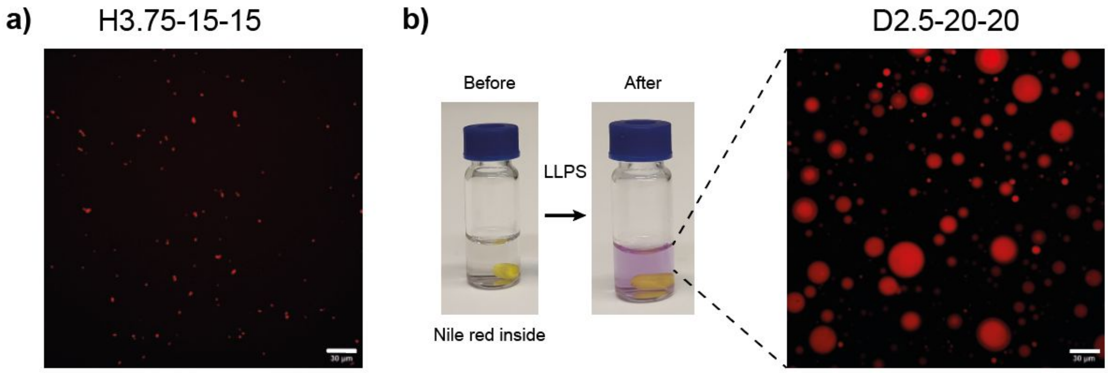

Figure S12. Microscopic morphology of H3.75-15-15 a) and D2.5-20-20 b) stained with 2.5 μM Nile red . Scale bar: 30 μm.

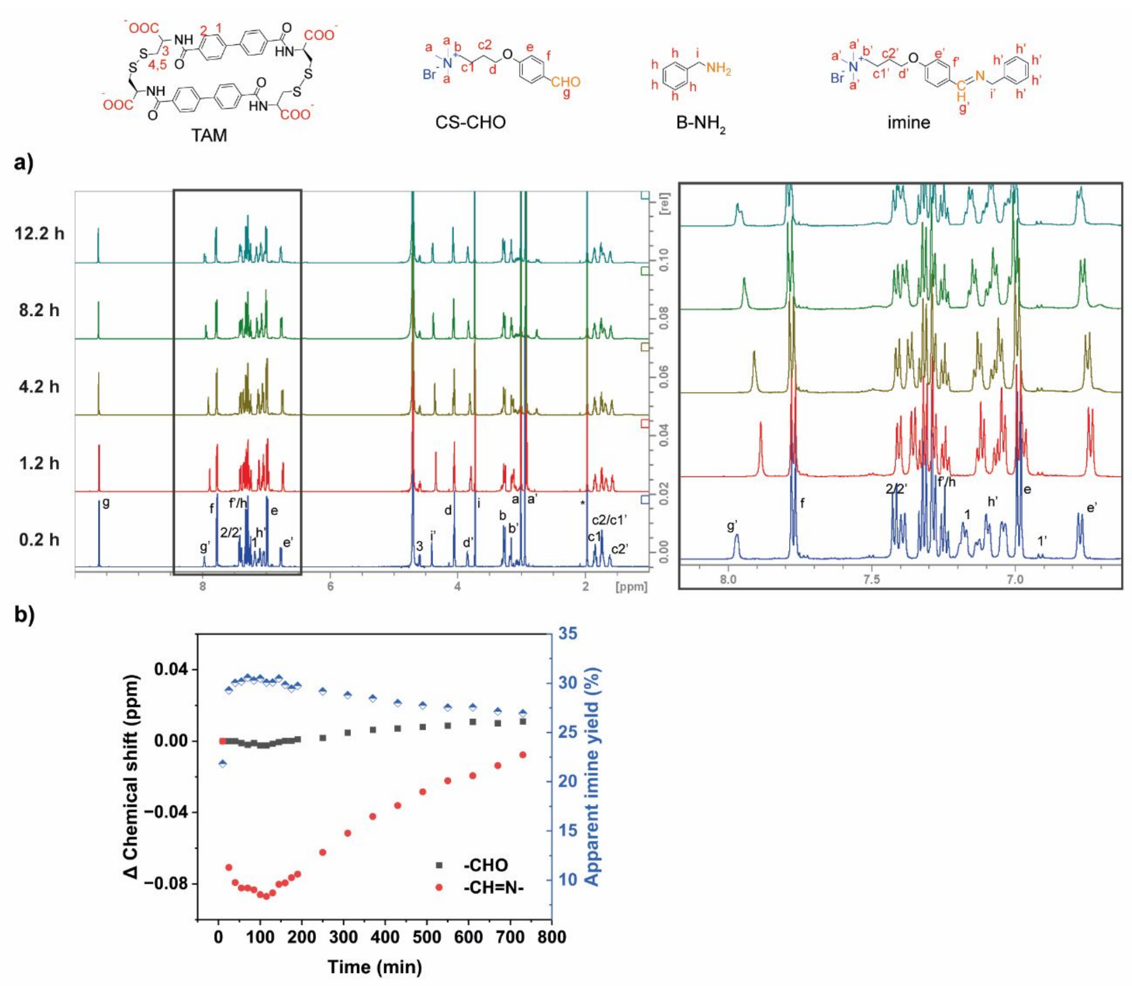

Figure S13. a) Time-dependent proton NMR spectra (500 MHz) of sample D2.5-20-20 with enlarged spectra (right panel) indicated in black box. b) Corresponding chemical shift changes for protons (-CHO and -CH=N-) relative to their initial positions, and the apparent imine yield over time, calculated from the integrals of imine-related peaks. *refer to the residual peak of CH_3CN. The apparent imine yield of D2.5-20-20 reached a maximum value of 30.5% in the first 40 minutes and then slowly decreased to 26.95%. The subsequent decline in imine yield was attributed to partial incorporation of imine into the phase-separated phase, indicated by broadened peak g'. Regarding the chemical shift change for imine proton in late-stage, this attenuation could be affected by the inhomogeneity caused by droplet sedimentation and coalescence in a NMR tube.

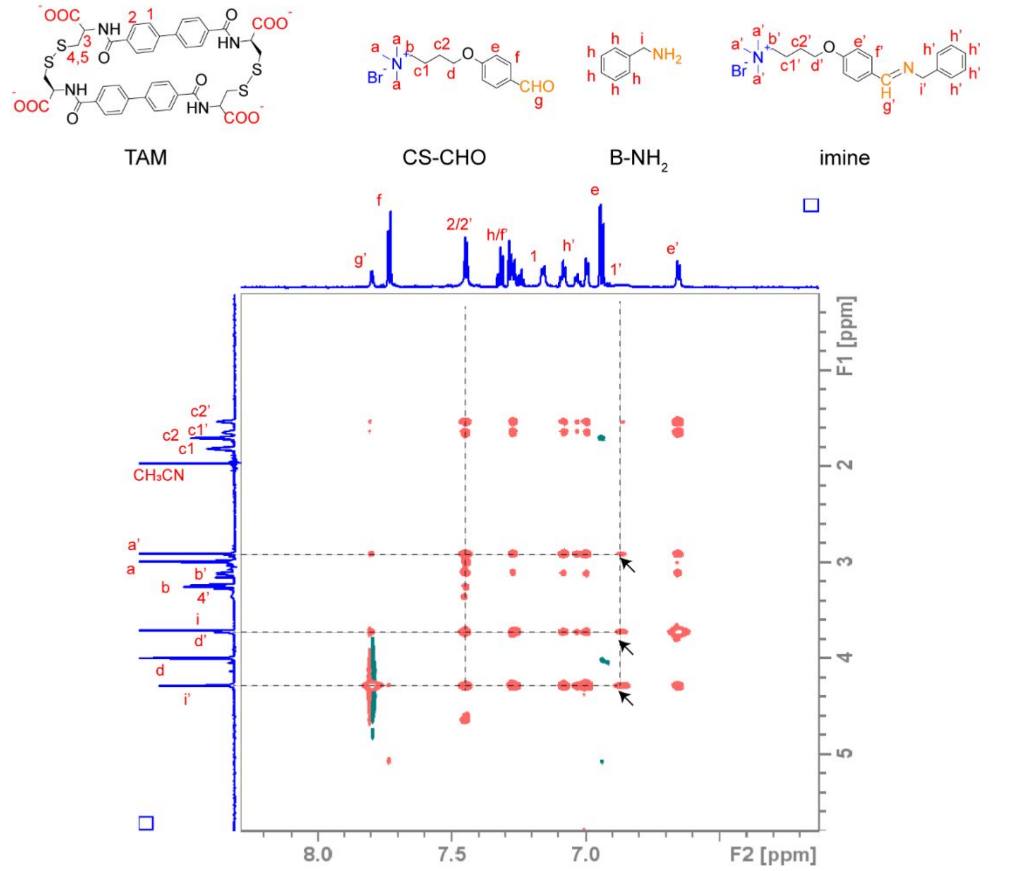

Figure S14. Two-dimensional nuclear Overhauser effect spectroscopy (NOESY) plot of sample D3.75-15-15 at 850 MHz.

a)

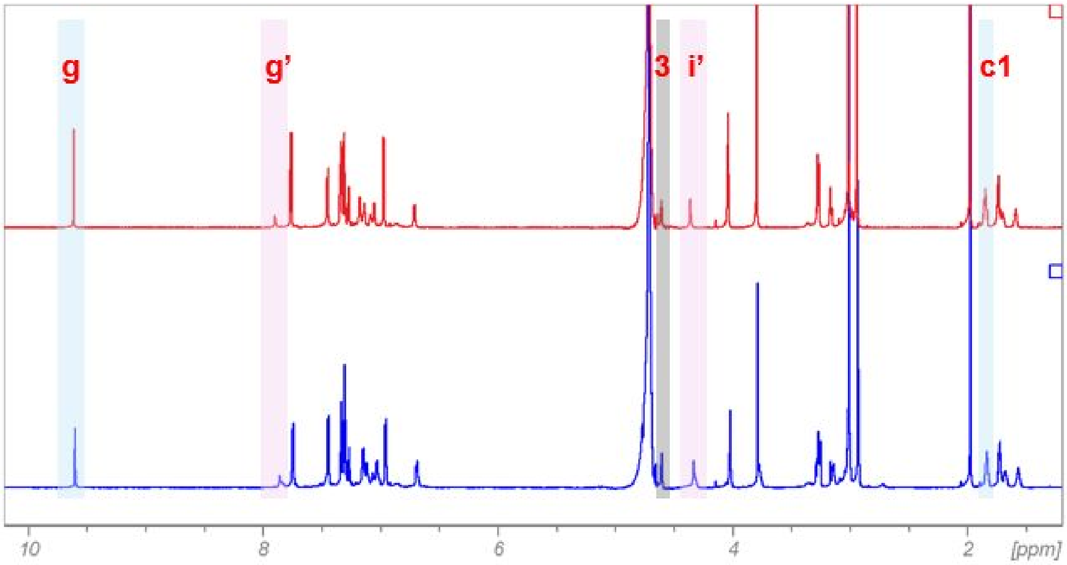

b)

Absolute integral	CS-CHO		Imine surfactant		TAM
	Peak g	Peak c1	Peak g'	Peak i'	Peak 3
Supernatant	170351.56	447411.97	33156.62	143663.19	128995.75
Original	160425.91	470546.09	62784.25	209507.19	167879.59
Change ratio	-6%	5%	47%	31%	23%

Figure S15. a) 1H NMR spectra (500 MHz) of supernatant of D3.75-15-15 after centrifugation (top, 1000 rpm for 30min) and of the original sample (bottom). b) Absolute integrals of selected proton signals in the supernatant and original D3.75-15-15, and their relative changes.

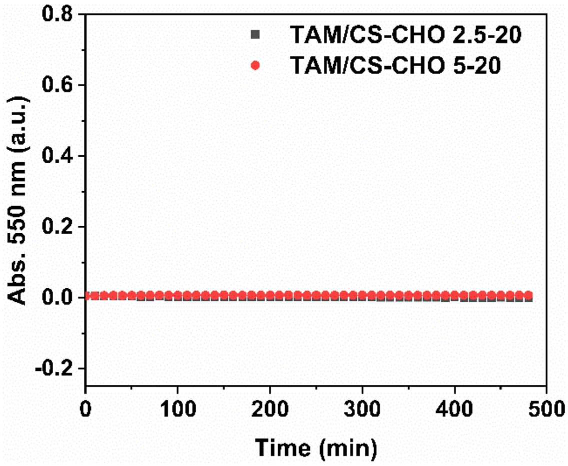

Figure S16. Turbidity tests of samples TAM/CS-CHO with concentration ratios of 2.5 mM : 20 mM or 5 mM : 20 mM in D_2O buffer.

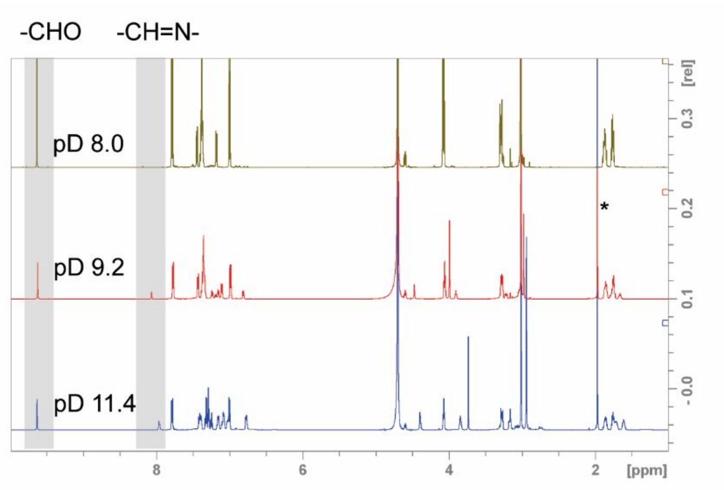

pD	imine yield (%)
8.0	none
9.2	11
11.4	27

Figure S17. Proton NMR spectra (500 MHz) of samples D2.5-20-20 with variable pD and imine yield analyzed from peak integration. The results shown that as the pD decreased from 11.4 to 8.0, the amount of produced imine decreased. *refer to the residual peak of CH_3CN.

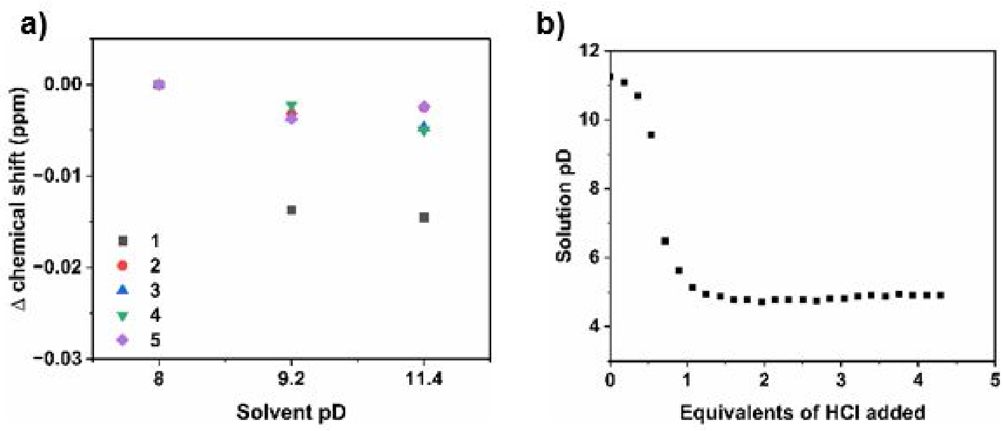

Figure S18. a) pD-dependent changes in proton chemical shifts of TAM (2.5 mM), relative to the sample at pD 8.0 (500 MHz). b) Acid-base titration curve of TAM obtained by titration with 0.1 M HCl.

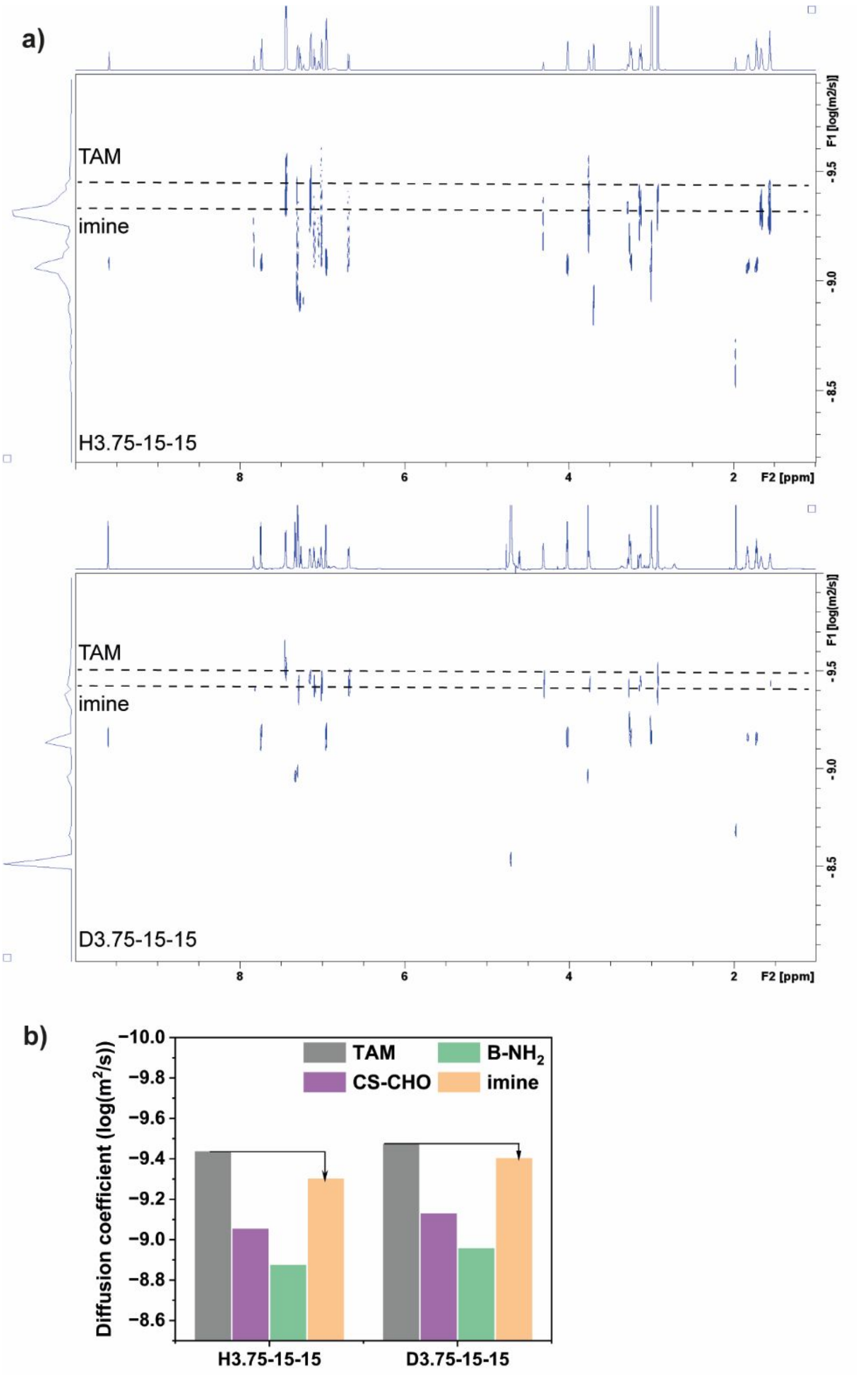

Figure S19. a) DOSY spectra of sample H3.75-15-15 and D3.75-15-15 at 850 MHz. b) Comparison of diffusion coefficients for components in H3.75-15-15 and D3.75-15-15. In D3.75-15-15, diffusion coefficient of imine (3.91×10^{-10} m^2/s) was close to that of TAM (3.33×10^{-10} m^2/s), while in H3.75-15-15 those diffusion coefficients of imine and TAM were 4.94×10^{-10} m^2/s and 3.62×10^{-10} m^2/s, respectively.

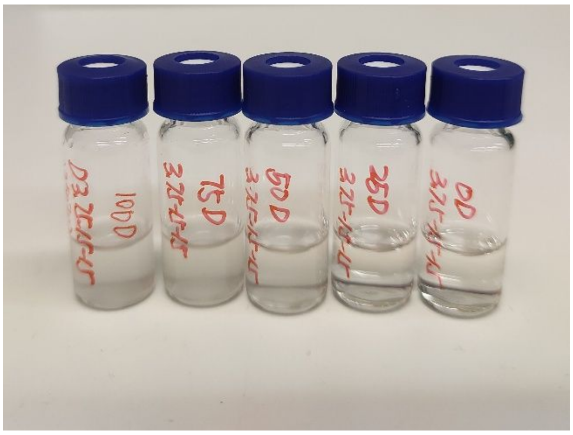

Figure S20. Equilibrated samples H-D 3.75-15-15 with varying D_2O content (100%, 75%, 50%, 25%, and 0%, from left to right) showing a monotonic decrease in turbidity.

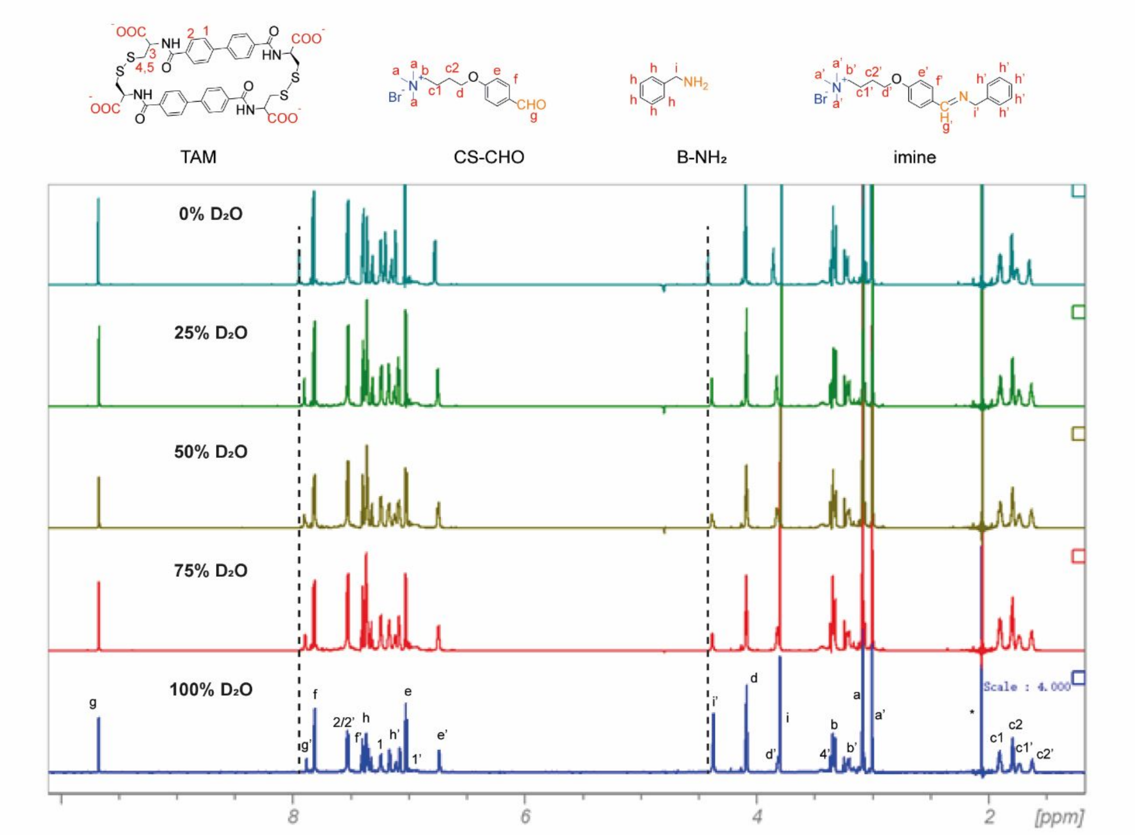

Figure S21. Proton spectra of samples H-D 3.75-15-15 with varying D_2O content at 600 MHz. The dashed line were drawn to assist in illustrating the chemical shift change for imine proton (peak g') and benzylic proton (peak i'), respectively. * refer to the residual peak of CH_3CN.

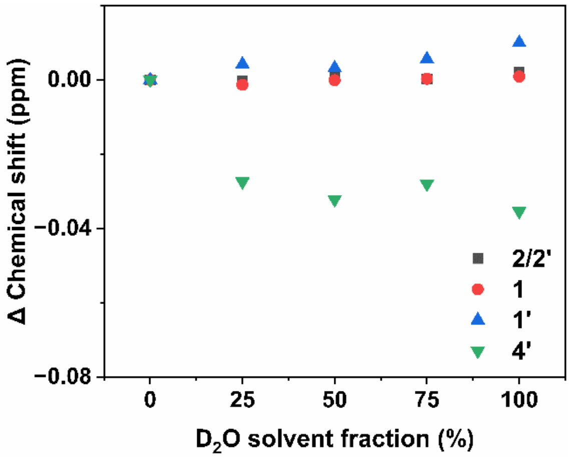

Figure S22. Changes in proton chemical shifts of anionic TAM from samples H-D 3.75-15-15 with varying D_2O fractions, relative to those in non-deuterated buffer. 1 and 1’ represented aromatic protons in a free state and assembled state, respectively. The similar notation also applied to 2 and 2’. Data was collected on a Bruker 600 MHz NMR spectrometer.

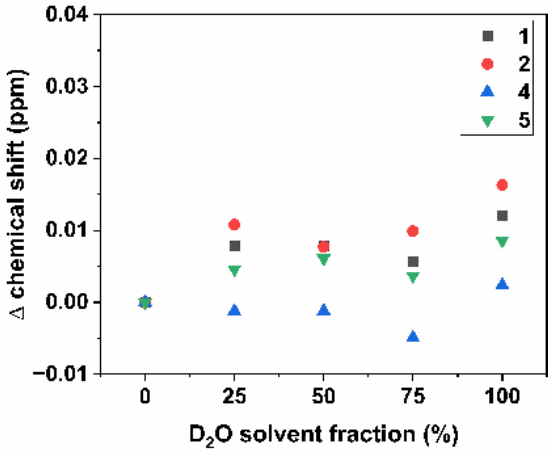

Figure S23. D_2O-dependent changes in the proton chemical shifts for TAM alone, relative to the 0% D_2O condition.

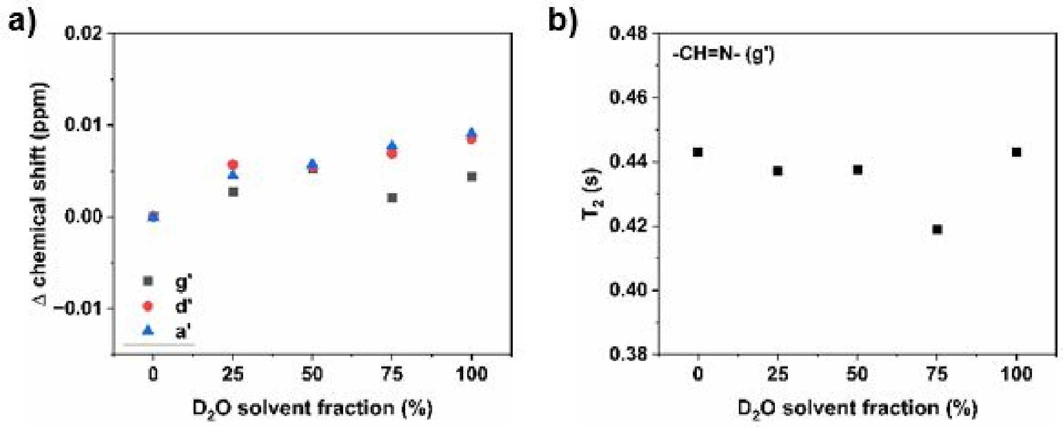

Figure S24. A) D_2O-dependent changes in the proton chemical shifts of cationic imine surfactant in samples H-D 0-15-15, relative to the 0% D_2O condition, b) Transverse relaxation time (T_2) of the imine proton (-CH=N-, peak g’) in H-D 0-15-15 samples as a function of D_2O fraction.

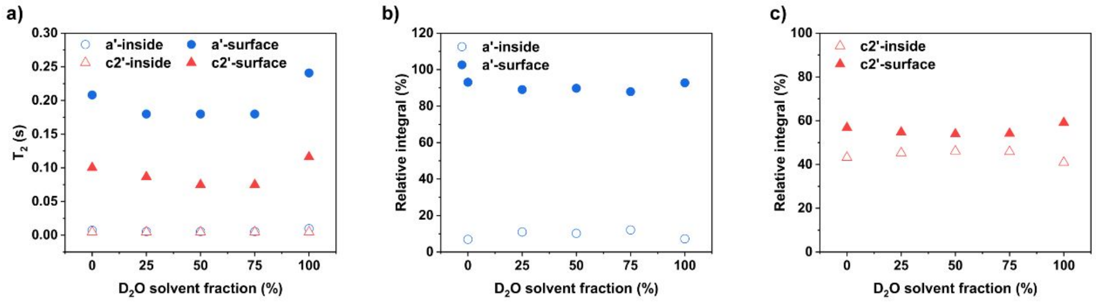

Figure S25. a) Transverse relaxation time (T_2) of selected imine surfactant protons (peak a' and c2') as a function of D_2O fraction, showing two motional populations. A dominant long T_2 was attributed to freely mobile protons, likely located on the surface of droplets, and a minor short T_2 was attributed to restricted protons, likely located in the inner layer. b) Relative integrals of two motional populations for quaternary ammonium proton (peak a'). c) Relative integrals of two motional populations for aliphatic proton (peak c2'). As quaternary ammonium proton (peak a') was located in the hydrophilic head of imine surfactant, the percentage of freely mobile population was evidently higher than that of restricted population. Moreover, as aliphatic proton (peak c2') was located in the middle position of imine surfactant, the percentage of restricted population was higher than the counterpart for quaternary ammonium proton. Data was collected on a Bruker 600 MHz NMR spectrometer.

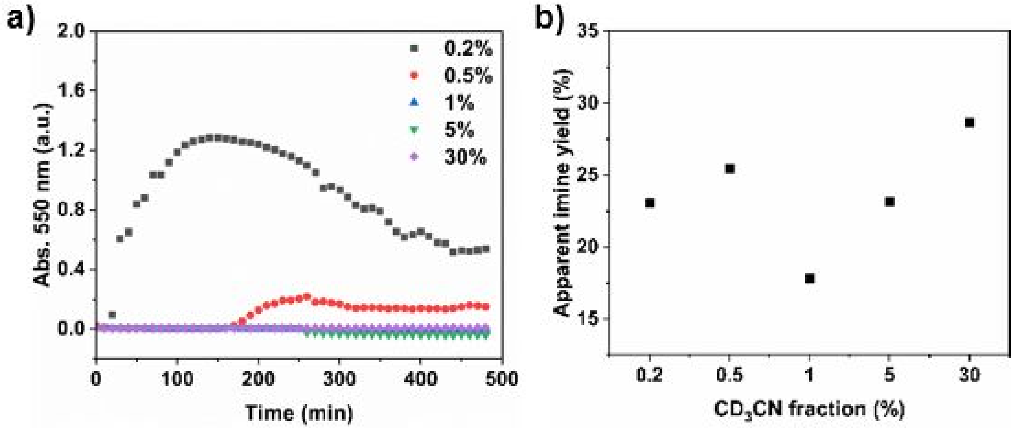

Figure S26. a) Time-dependent turbidity of D2.5-20-20 at different fractions of deuterated acetonitrile and b) the corresponding apparent imine yield determined by 1H NMR (500 MHz).

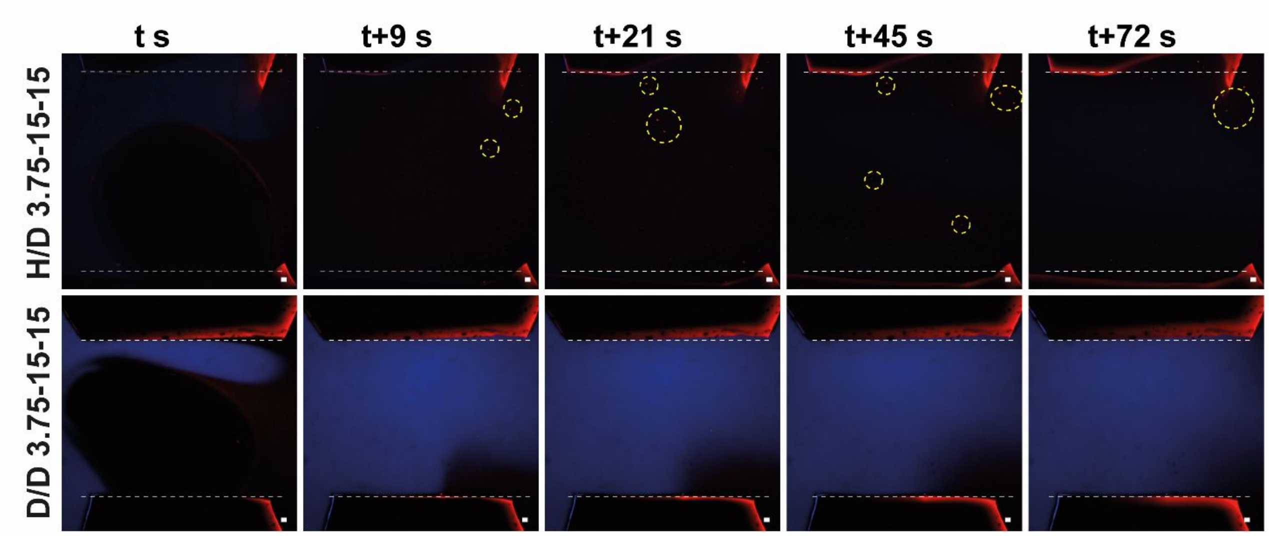

Figure S27. Snapshots from a time-lapse imaging session in the connecting tunnel where fluids from left and right side converged. H/D3.75-15-15 denoted that H3.75-15-15 (no LLPS) and D3.75-15-15 (LLPS droplets) were placed in the left and right well, respectively. D/D3.75-15-15 denoted that both wells were filled with D3.75-15-15. Dashed line indicated the tunnel boundaries, and dashed circle highlighted the moving droplets. Scale bar: 30 μm.

Table S1. Summary of sample states for DACA/CS-CHO/B-NH_2, DNCA/CS-CHO/B-NH_2, and DPCA/CS-CHO/B-NH_2 and TAM/CTAB systems in D_2O and H_2O buffers.

	D_2O			H_2O	
DACA/CS-CHO/B-NH_2	2.5-20-20	5-20-20	DACA/CS-CHO/B-NH_2	2.5-20-20	5-20-20
	1.875-15-15	3.75-15-15		1.875-15-15	3.75-15-15
DNCA/CS-CHO/B-NH_2	2.5-20-20	5-20-20	DNCA/CS-CHO/B-NH_2	2.5-20-20	5-20-20
	1.875-15-15	3.75-15-15		1.875-15-15	3.75-15-15
DPCA/CS-CHO/B-NH_2	2.5-20-20	5-20-20	DPCA/CS-CHO/B-NH_2	2.5-20-20	5-20-20
	1.875-15-15	3.75-15-15		1.875-15-15	3.75-15-15
TAM/CTAB	2.5-20	5-20	TAM/CTAB	2.5-20	5-20
	1.875-15	3.75-15		1.875-15	3.75-15

CTAB, cetyltrimethylammonium bromide; DACA, azobenzene-linked tetracarboxylic acid macrocycle; DNCA, naphthyl-linked tetracarboxylic acid macrocycle; DPCA, tphenyl-linked tetracarboxylic acid macrocycle; Pink boxes indicate phase separated-droplets; Blue boxes indicate irregular aggregate; Green boxes indicate single-phase

solution.

Below are Chemical structures of azobenzene-, naphthyl-, and phenyl-linked tetracarboxylic acid macrocycles, denoted by DACA, DNCA, and DPCA, respectively.

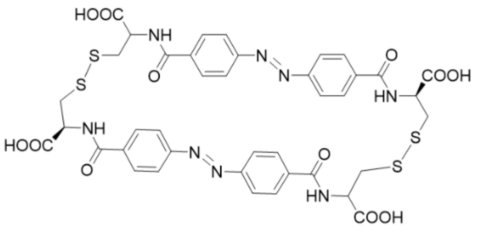

DACA

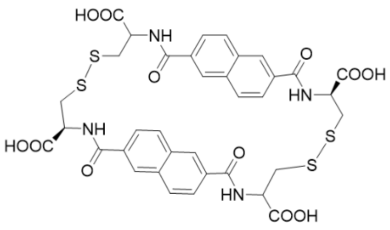

DNCA

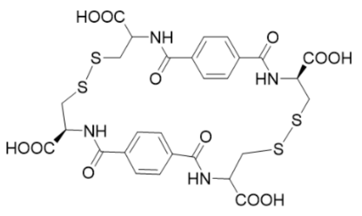

DPCA

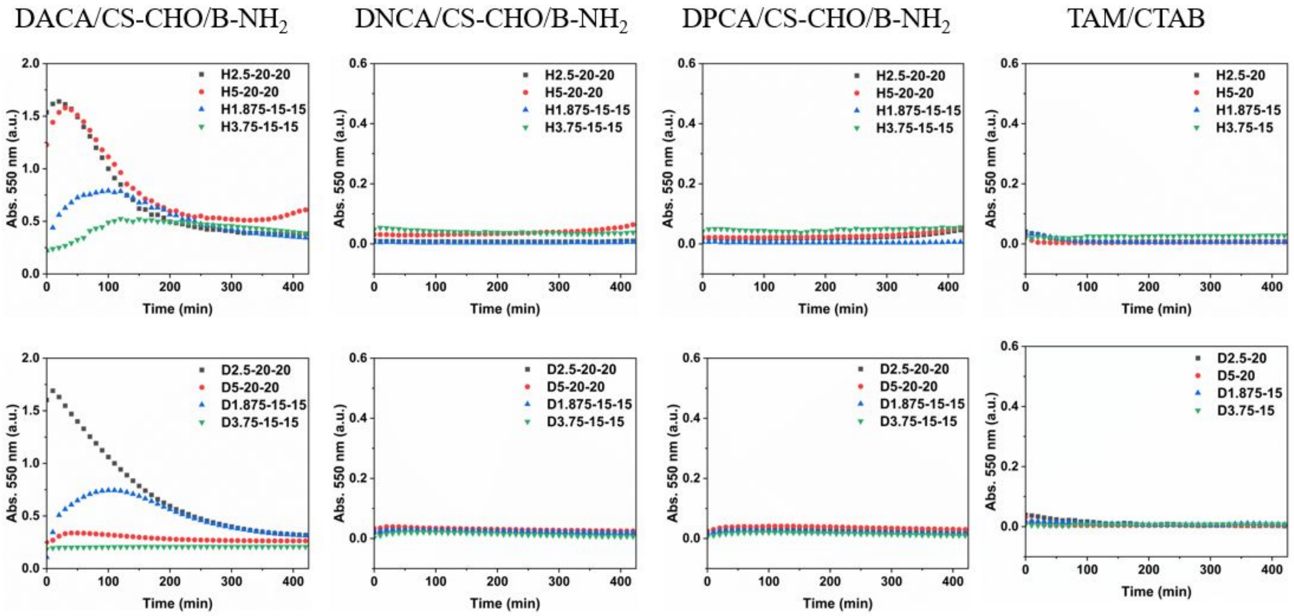

Figure S28. Time-dependent turbidity of DACA/CS-CHO-B-NH_2, DNCA/CS-CHO-B-NH_2, DPCA/CS-CHO-B-NH_2 and TAM/CTAB system at different composition ratios in H_2O (top) and D_2O (bottom) buffer, recorded with Cytation 5.

3. References

1. Kulchat, S.; Chaur, M. N.; Lehn, J.-M., Kinetic Selectivity and Thermodynamic Features of Competitive Imine Formation in Dynamic Covalent Chemistry. *Chemistry – A European Journal* **2017,** *23* (46), 11108-11118.

2. Yu, J.; Qi, D.; Mäkilä, E.; Lassila, L.; Papageorgiou, A. C.; Peurla, M.; Rosenholm, J. M.; Zhao, Z.; Vallittu, P.; Jalkanen, S.; Jia, C.; Li, J., Small-Molecule-based Supramolecular Plastics Mediated by Liquid-Liquid Phase Separation. *Angewandte Chemie International Edition* **2022,** *61* (39), e202204611.

3. Telkki, V.-V.; Urbańczyk, M.; Zhivonitko, V., Ultrafast methods for relaxation and diffusion. *Progress in Nuclear Magnetic Resonance Spectroscopy* **2021,** *126-127*, 101-120.

4. Telkki, V.-V., Hyperpolarized Laplace NMR. *Magnetic Resonance in Chemistry* **2018,** *56* (7), 619-632.

5. Minkenberg, C. B.; Li, F.; van Rijn, P.; Florusse, L.; Boekhoven, J.; Stuart, M. C. A.; Koper, G. J. M.; Eelkema, R.; van Esch, J. H., Responsive Vesicles from Dynamic Covalent Surfactants. *Angewandte Chemie International Edition* **2011,** *50* (15), 3421-3424.

6. Späth, F.; Donau, C.; Bergmann, A. M.; Kränzlein, M.; Synatschke, C. V.; Rieger, B.; Boekhoven, J.,

Molecular Design of Chemically Fueled Peptide–Polyelectrolyte Coacervate-Based Assemblies. *Journal of the American Chemical Society* **2021,** *143* (12), 4782-4789.
